# Multiplexing antibiotic screening assay in droplet microfluidics

**DOI:** 10.1038/s41598-026-55537-2

**Published:** 2026-06-03

**Authors:** Sundar Hengoju, Karin Martin, Kirstin Scherlach, Martin Roth, Miriam A. Rosenbaum

**Affiliations:** 1 Bio Pilot Plant, Leibniz Institute for Natural Product Research and Infection Biology – Hans-Knöll-Institute, 07745 Jena, Germany; 2 Biomolecular Chemistry, Leibniz Institute for Natural Product Research and Infection Biology – Hans-Knöll-Institute, 07745 Jena, Germany; 3 Faculty of Biological Sciences, Friedrich Schiller University Jena, 07743 Jena, Germany

**Keywords:** High-throughput phenotypic assay, Natural product discovery, Whole-cell reporter strains, Picolitre cultivation, Biological techniques, Biotechnology, Microbiology

## Abstract

Environmental samples contain complex microbial communities hiding a treasure trove of active compounds. However, screening for active natural products from environmental samples is challenging due to inefficient cultivation techniques and a lack of proper screening platforms. For empowering antibiotic screening assays from complex microbial communities, we have developed and optimized a droplet-based platform with multiplexing capability. A cultivation strategy for bacteria in picoliter droplets was combined with phenotypic screening using multiple whole-cell reporter species. A mixture of two different fluorescently labelled reporter strains, one Gram-positive and one Gram-negative, was picoinjected to each of millions of picoliter cultures, which were screened for inhibiting activity based on the independent survival signals of each reporter species. Proof-of-concept experiments demonstrate efficient detection, selection, and recovery of a model *Streptomyces* strain from a synthetic mixture according to the specific inhibition of the reporter strains by the produced antibiotic. Subsequently, the established platform was successfully applied to screen environmental microbial communities from soil samples. This approach showcases multiplexing capabilities for screening assays in microfluidic droplets in order to simultaneously screen for new bioactive compounds with various inhibition profiles.

**Supplementary Information:**

The online version contains supplementary material available at 10.1038/s41598-026-55537-2.

## Introduction

The rise in antimicrobial resistance (AMR) has been recognized as a serious threat to global public health. According to recent statistics, AMR surveillance for selected bacteria showed a significant increase in the emergence of dangerous resistant pathogens^1–4^. After the golden era of antibiotic discovery, no new types of antibiotics were reported causing a void in the antibiotic pipeline^5^. So, the rapid emergence of resistance to known antibiotics, while no new ones are being discovered, has resulted in an urgent need for antibiotics with a novel mode of action.

To fill the antibiotic discovery void, many research and pharmaceutical companies focused on developing synthetic antibiotics using in vitro screening of synthetic chemical libraries against a particular, or sometimes hypothetical target^6^. It was thought that chemicals inhibiting a single enzyme/target of a pathogen in vitro would also show potent activity against infection scenarios. However, due to ineffective level of bacterial cell penetration, chemical instability, toxicity, and chemical degradation/elimination from the body, among other issues, the hope for molecules obtained in these targeted screenings faded soon^6–9^. Although several inhibitors for different targets were identified, not a single drug with a reasonable spectrum of activity against pathogens was discovered by applying this platform^10^.

Natural products are historically successful and still a viable source for new antibiotics. Based on the traditional systematic approach developed by Waksman with whole-cell phenotypic screening, the majority of currently used antibiotics were found during the golden era of antibiotic discovery between the 1940s and 1960s. Whole-cell phenotypic screening allows identification and selection of active compounds that are capable of permeating cell wall and affecting the target. Recently, natural product screening has been preferred over synthetic compound screening both for the superior diversity of available compounds and the fact that natural products have already been pre-screened by natural evolution^7,11–13^. With the revival of the Waksman approach, exploration of microorganisms from various ecological niches has recently identified many promising microbial species with possibilities for antibiotic activity^13,14^. This has been further proved with the recent discovery of darobactin^15^ and teixobactin^16^ from a soil sample. And yet, due to the enormous diversity and complexity of the samples, many natural products are still undiscovered.

Natural samples contain a tremendous microbial diversity with an enormous potential of active compounds. It has been estimated that at least 10^7^ strains must be screened for discovering a novel active product^8^. For screening such large samples, high-throughput screening platforms with the capability of cultivation and analysis of millions of microbial isolates are required. The use of microtiter plates with robots for liquid handling has allowed growth and analysis of many microorganisms. However, this technology is limited to hundreds or thousands of strains and conditions, while resulting in huge costs and media consumption^17^.

Droplet microfluidics allows high-throughput experimentation by miniaturization, compartmentalization, and parallelization of reaction vessels^18–20^. Droplets of an aqueous phase, with volumes ranging from few picoliters to nanoliters, are generated in a continuous oil phase at rates often exceeding 1000/s. This allows the analysis of millions of samples in a short time. Furthermore, it allows flexibility in fluidic operations including splitting of droplets, addition of reagents, and sorting. Droplet microfluidics has been demonstrated for various microbiological cultivations including *Actinobacteria*^14,21^, soil samples^13^, marine samples^22^, bear microbiota^23^, mouse microbiome^24^, and human gut bacteria^25^.

Furthermore, droplet microfluidics enables culturing the slow-growing and up to now unculturable microorganisms^13,25–27^. This is possible by stochastically confining single microbial cells in droplets as separate tiny liquid compartments^18,24,28^. The confinement prevents nutrient and space competition among slow- and fast-growing microbes and allows slow-growing microbes to grow freely on their own. With knowledge of only about 1% of microbes being cultivated to date^29,30^, droplet microfluidics opens the door for domesticating millions of slow-growing and uncultured microbial species^13,31^. These newly accessible microorganisms, up-concentrated inside of droplets, provide an ideal opportunity to detect novel natural products.

However, the use of droplet microfluidics for antibiotic screening is still limited due to the inefficient detection of inhibiting activity and the lack of multiplexing capability. For bioactivity-based phenotypic screening, only a single reporter strain is utilized^14,23^, which limits the screening for active compounds since only molecules inhibiting the selected reporter strain can be detected. This restricts the possibility of finding novel bioactive molecules having anti-microbial activity against other pathogens. These assays also do not provide any information about the specificity of molecules with regard to Gram-positive and Gram-negative pathogens. Furthermore, these conventional screening approaches are based on fluorescence microscope setups, which are complicated, tedious, and expensive, limiting the multiplexing capability and wide application of droplet microfluidics for antibiotic screening^32^.

**Fig. 1:**
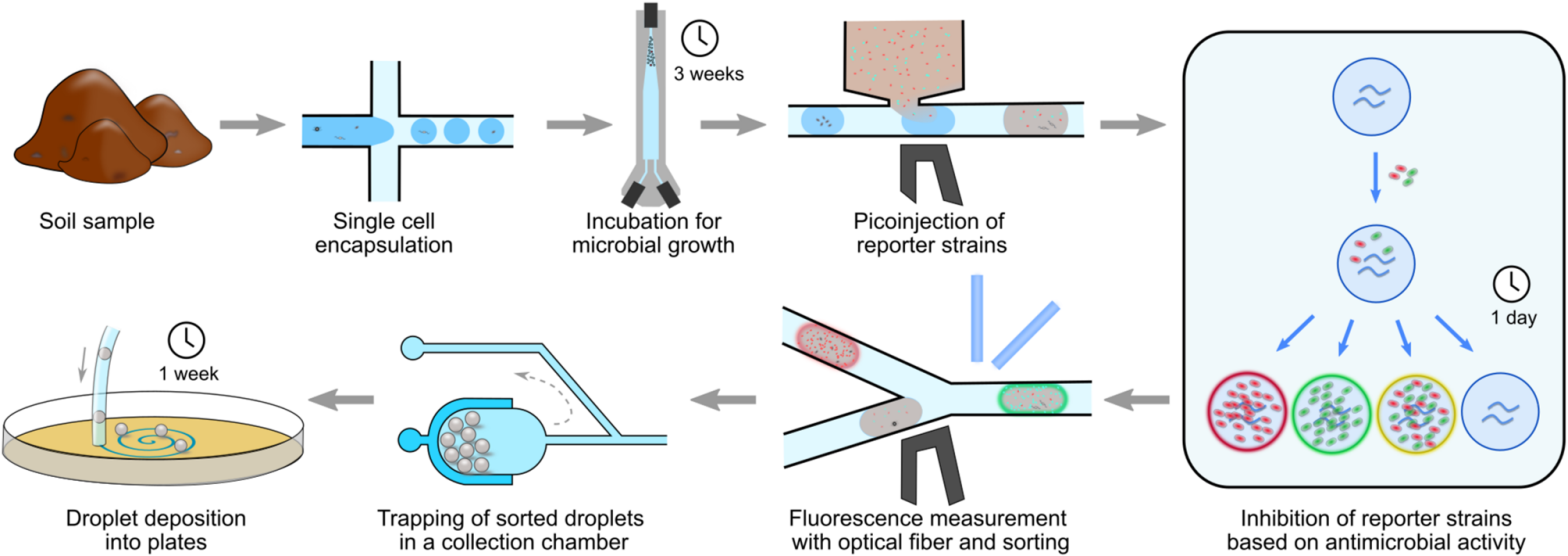
General workflow of the multiplexed antibiotic screening platform using an optofluidic setup. Incubation times are presented for droplet growth of environmental microbes, reporter strains inhibition testing, and recovery on agar plates.

Here, we present an optimized droplet microfluidic strategy for multiplexing Gram-positive and Gram-negative reporter strains in the antibiotic screening with an integrated optical fiber-based detection setup (Fig. 1). A previously developed technique for culturing microbes in droplets^33^ is combined with multi-color fluorescence detection using optical fibers^34^. Two reporter strains are simultaneously added to droplets containing cultures of antibiotic producing bacteria and antibacterial activities are tested based on the whole-cell phenotypic assay. The applicability of our workflow is demonstrated with highly diverse microbial populations derived from different soil samples.

## Results

### Selection of reporter strains

**Fig. 2:**
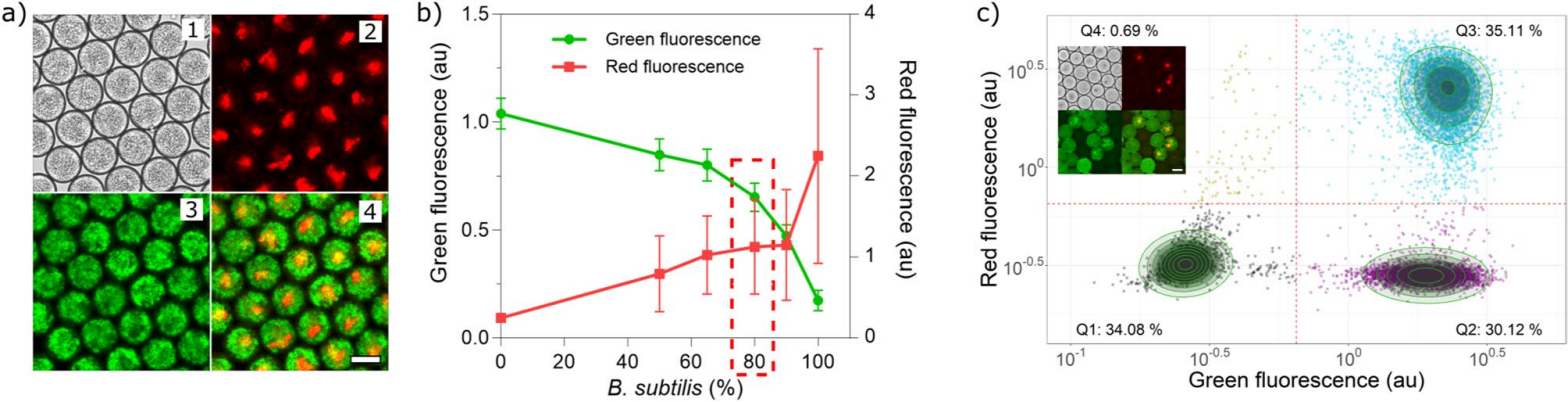
Selection of reporter strains. (**a**) Two reporter strains, *E. coli* (EC081) and *B. subtilis* (BS168) constitutively expressing GFP and mKate, respectively, were co-cultured in droplets. The growth of reporter strains was determined by measuring fluorescence intensities in the green and red channels from images. 1-brightfield, 2-red, 3-green, and 4-overlay of green and red. (**b**) Green and red fluorescence intensities of droplets generated with different inoculum ratios were measured using the optofluidic platform. The X-axis represents the concentration of *B. subtilis* BS168 in inoculum mixtures. Optimal fluorescence intensities in both green and red channels were found at a 20:80 inoculation ratio of *E. coli* EC081 and *B. subtilis* BS168 (dashed rectangle). (**c**) Red and green fluorescence intensities to indicate growth or inhibition of the reporter strains are depicted for droplets after 24 h co-incubation. Low red/low green droplet sub-population indicating presence of *S. noursei* as antibiotic producer inhibiting both reporter strains (lower left quadrant, black-labeled), low red/high green droplet sub-population indicating *S. hygroscopicus* as antibiotic producer inhibiting only one reporter strain (lower right quadrant, purple-labeled, i.e., inhibiting *B. subtilis*) and high red/high green droplet sub-population indicating droplets without any *Streptomyces* strains as a negative control (upper right quadrant, blue-labeled). Each data point represents a droplet. Red dotted lines represent the thresholds for red and green fluorescence for classifying droplets into different groups. The relative frequency within each quadrant is specified at corners. Scale bar is 50 μm.

For multiplexing the antibiotic screening assay, we utilized two reporter strains, one Gram-positive and one Gram-negative. We selected reporter strains expressing different complementary fluorescent proteins. The *Escherichia coli* strain (EC081) constitutively produces the green fluorescent protein GFP, and the *Bacillus subtilis* strain (BS168) is constitutively synthesizing the red fluorescent protein mKate. Initially, we tested co-cultures of the reporter strains in a ratio of 1:1 for evaluating growth competition. Growth and fluorescence intensities in green and red channels were measured. We observed neither inhibitory nor synergistic effects (Figs. 2a and S1). We then cultured the reporter strains at different inoculum ratios in well plates in the microcultivation platform BioLector II (Fig. S2), to check the effect of initial bacterial cell concentrations. Increasing amount of fluorescence was observed depending on initial inoculum concentration. Additionally, after 24 h incubation in well plates, droplets were generated and fluorescence intensities were measured using the optofluidic platform^34^. An inoculum ratio of 20:80 of *E. coli* GFP (EC081) and *B. subtilis* mKate (BS168) resulted in an optimal increment of both red and green fluorescent signals (Fig. 2b). Thus, this ratio was applied for further experimentation.

### Detection of active compounds in droplets

We initially performed multiplexed inhibition assays with mixed reporter strains (20:80 ratio of *E. coli* EC081 and *B. subtilis* BS168) using supernatants of different *Streptomyces* in microtiter well plate and confirmed selective antimicrobial activity against our reporter strains (Fig. S4). For droplet assays, we generated three different droplet populations: (1) droplets containing spores of a *Streptomyces* strain (i.e., *S. noursei*) producing an antimicrobial metabolite effective against both reporter stains (EC081 and BS168), (2) droplets containing spores of a *Streptomyces* strain (i.e., *S. hygroscopicus*) producing an antimicrobial metabolite effective against only one reporter strain (only affecting the red-fluorescing *B. subtilis* BS168), and (3) droplets without any spores but only medium. Droplets of the three populations were mixed in equal proportion (one third each) and incubated for 4 days. Simultaneously, the three individual droplet populations were also incubated in three separate incubation chambers as control droplets. After 4 days of incubation, dense *Streptomyces* mycelia were grown in inoculated droplets (Fig. S5). A mixture of our two reporter strains was added into all droplets with an optimized picoinjection process (Supp info Picoinjection process, Fig. S21 and Video S1) and droplet incubation was continued for another 24 h. Afterwards, signal intensities from droplets were measured by using the optofluidic setup.

Initially, red and green fluorescent intensities were measured from the control droplets incubated separately in different incubation chambers. These intensities were used for categorizing droplets of the mixed population into 3 different groups: (1) droplets with both reporter strains inhibited, (2) droplets with only the red *B. subtilis* reporter strain inhibited, and (3) droplets with proliferating reporter strains. From the subsequent analysis of the mixed droplet population, ~ 34.08% of droplets had low red and low green fluorescence intensities as in the first control droplet population, which inhibited both reporter strains. Similarly, ~ 30.12% of droplets had low red and high green fluorescence intensities, which inhibited only the red *B. subtilis* reporter strain while ~ 35.11% of droplets had high red and high green fluorescence intensities, with no inhibition effect (Fig. 2c). Furthermore, a similar ratio for the classification of a mixed population was also obtained by image analysis of mixed and control droplet populations with highly overlapping clusters in an overlay plot (Fig. 2c, inset and Fig. S6). This result demonstrates a good correlation of inhibitory activity of *Streptomyces* when cultivated in a mixed droplet population and in conventional microtiter well plates (Fig. S4). Hence, this result serves as a proof-of-principle for the selective detection of active compounds in droplets.

### Screening a model library of *Streptomyces*

**Fig. 3:**
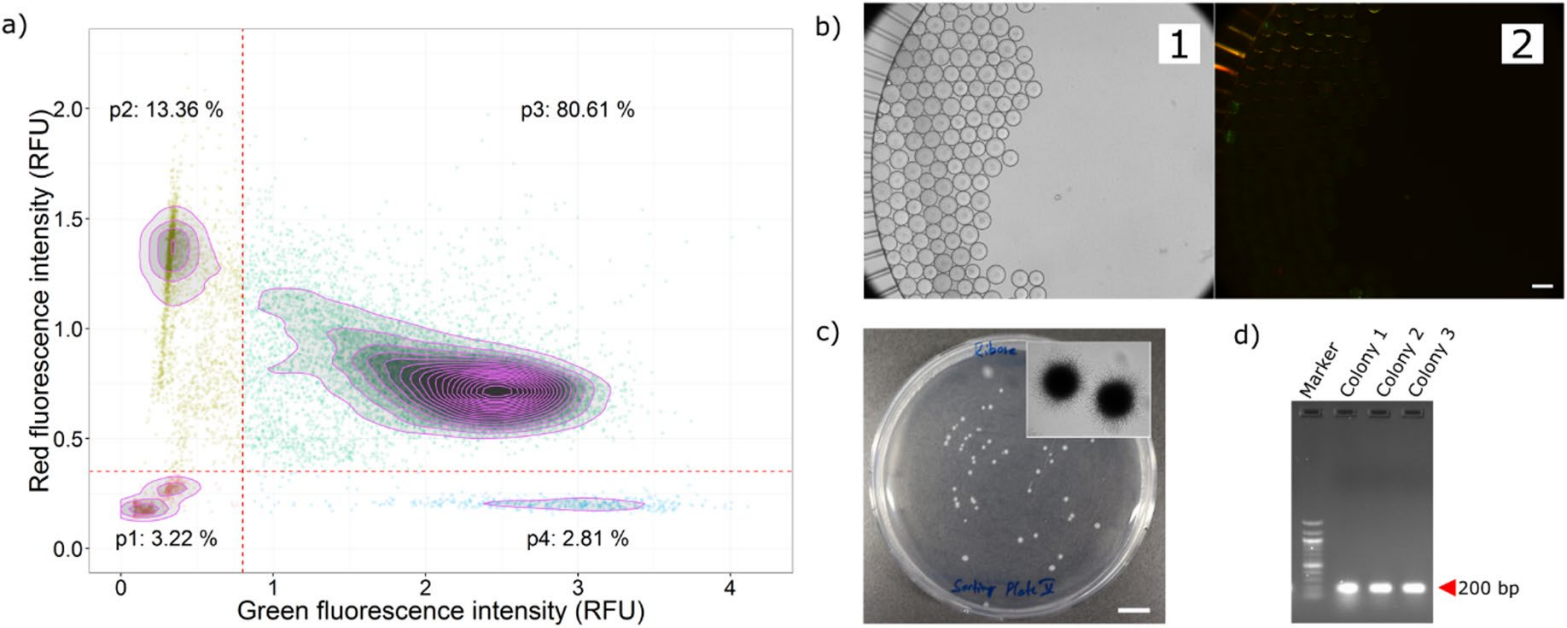
Inhibition of reporter cells by an artificial library of five different *Streptomyces* strains. (**a**) Droplets of the library strains were picoinjected with a mixture of the *E. coli* GFP and *B. subtilis* mKate reporter strains. Red and green fluorescence intensities for droplets after 24 h co-incubation are depicted. Each data point represents a droplet. Red dotted lines represent the thresholds for red and green fluorescence for classifying droplets into different groups. The relative frequency within each quadrant is specified at corners. (**b**) Image of sorted droplets in the collection chamber. 1-bright-field, 2-overlay of green and red fluorescence images. (**c**) Agar plate with colonies grown from deposited droplets. Typical colony morphology with hyphae is shown in the inset image. (**d**) Analysis of amplified gene products for the detection of the *S. noursei* strain-specific nourseothricin acetyltransferase gene from deposited colonies. In total, 49 colonies were analyzed. More than 86% of recovered colonies showed bands at the expected length. Results of control experiments with all employed *Streptomyces* strains are shown in Suppl. Info. Fig. S11. Scale bars are 100 μm for (**b**) and 1 cm for (**c**).

As a model antibiotic producer library, we prepared an artificial mixture of spores of five different *Streptomyces* strains with different antibiotic production profile in equal proportion (Table S2). One of the strains, *S. noursei* produces the streptothricin antibiotic nourseothricin, which is known and was confirmed to inhibit both reporter strains (Fig. 2 and Fig. S7). This strain was used as the target selection strain during this screening process. The other four strains inhibited only one or none of our reporter strains (Table S2). Droplets were generated targeting single spores per droplet (Poisson distribution at λ = 0.4). After incubation for growth, a mixture of both reporter strains (at 20:80 ratio of *E. coli* EC081 and *B. subtilis* BS168) was picoinjected into the droplets and incubated for another 24 h (Fig. S8). Fluorescence signals from droplets were detected and analyzed. In the library, 3.22% of droplets had low red and low green fluorescence intensities reflecting inhibition of both reporter strains (Fig. 3a). Similarly, we found 2.81% and 12.36% of droplets with either only low red or only low green fluorescence intensities, respectively. These populations demonstrate inhibition of only one reporter strain (low red: *B. subtilis* inhibited and low green: *E. coli* inhibited) indicating potential producers of active compounds with selective activity.

Droplets inhibiting both reporter strains were actively sorted using an optimized droplet sorting process (Supp info Sorting setup and electronics, Fig. S22 and Video S2). Droplets were sorted into the collection structure and later deposited onto agar medium on Petri dishes (Supp info Recovery of droplets, Fig. S23 and video S3). The microscopic analysis confirmed that all sorted droplets had low green and low red fluorescence intensities, indicating successful active sorting (Fig. 3b). After sorted droplets were deposited onto agar medium and incubated, all grown colonies were verified by observing colony morphology under microscopes (Figs. 3c and S9). Most of the colonies showed hyphae structures and spores in microscopic images, demonstrating the growth of sorted filamentous *Streptomyces* strains. A few colonies of clearly differentiable non-filamentous reporter strains were also observed, which might be due to two reasons. Firstly, the reporter strains in some droplets might not be completely inhibited (i.e. just inhibited but not killed) due to a low amount of produced active compound, which is relatable to growth variabilities of the producers. Secondly, once these droplets were deposited onto agar plates, the inhibitory activity in the confined droplet is lost as the droplet breaks and the antimicrobial compound is diffusing into the medium. The higher growth rate of the reporter strain favours its growth rather than the *Streptomyces* strain. One solution to reduce the growth of reporter strains would be to use selective media for streptomycetes. Several agar media were tested for enhanced growth of *Streptomyces* (Supp Info Selection of agar media and Fig. S10). Agar medium with ribose as carbon source showed satisfactory growth of the streptomycetes with lower number of reporter strain colonies and was selected for this experiment. From deposited and grown colonies on ribose-agar medium, 50 colonies were randomly picked and tested for the presence of the nourseothricin acetyltransferase gene, which is specific for the target strain *S. noursei*, by performing PCR with custom-designed primers (Supp Info Validation of sorted droplets and Fig. S11). Analysis of the amplified gene length revealed that 86% of colonies belonged to the target strain (Figs. 3d and S12) whereas the remaining colonies could belong either to reporter strains or other *Streptomyces* strains. It has to be noted, that the actual droplet sorting process was highly efficient as no fluorescence is observed from sorted droplets (Fig. 3b).

### Screening of environmental samples

**Fig. 4:**
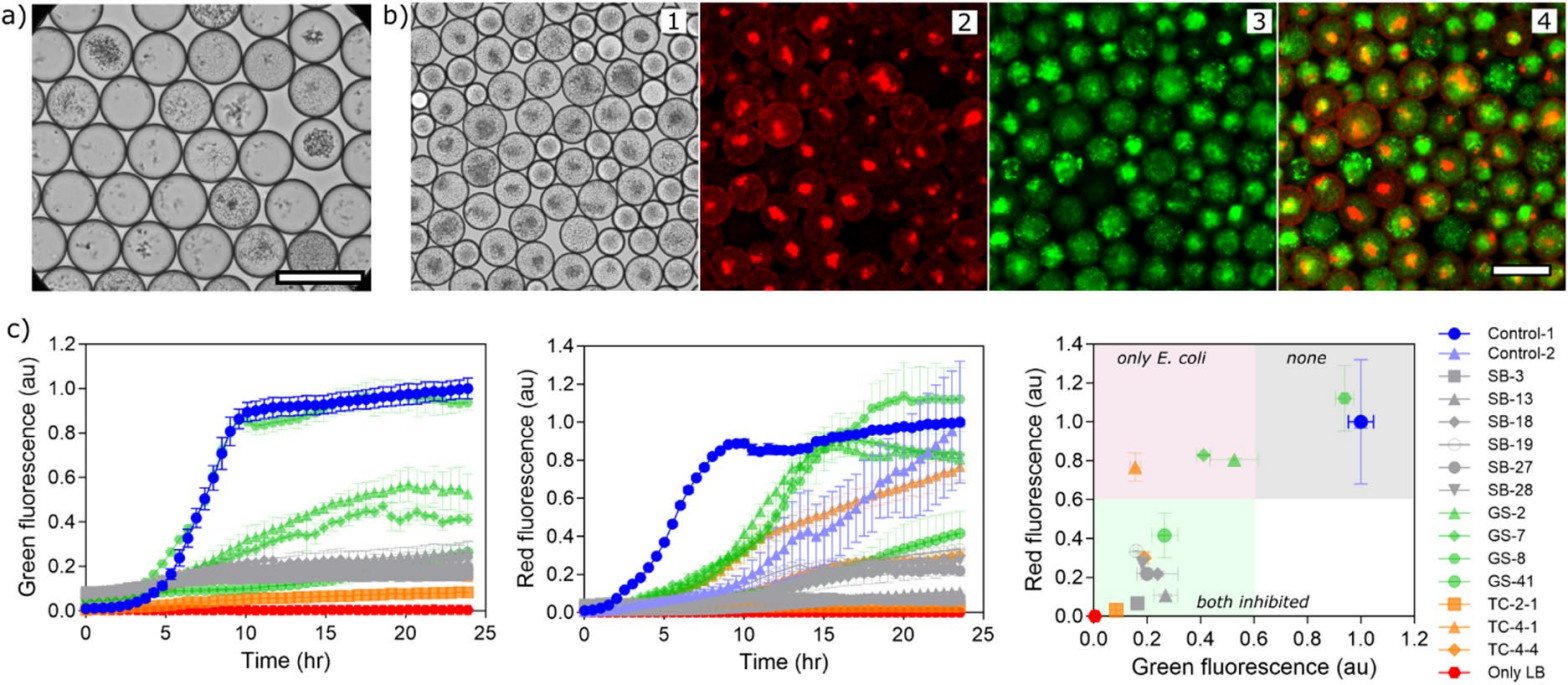
Multiplexed inhibition assay with three different environmental soil samples. (**a**) Incubated droplets with microorganisms from soil showing growth with diverse morphologies. (**b**) A mixture of both reporter strains – green *E. coli* GFP and red *B. subtilis* mKate – was added into droplets and fluorescence intensities were measured by imaging or optofluidic setup for sorting. 1-bright-field, 2-red, 3-green, 4-overlay of green and red fluorescence images. Droplets with inhibitory activity were sorted and strains were isolated. (**c**) Validating inhibition of reporter strains by cell suspensions of recovered isolates in microtiter well plates. Growth of reporter strains was quantified by monitoring green (for *E. coli* EC081, left-panel) and red (for *B. subtilis*, center-panel) fluorescence signals. *B. subtilis* strain BS168 was used to test isolates from the SB sample (colored grey), while strain BS3610 was used for GS (colored light green) and TC (colored light orange) samples. Wells containing growth media with reporter strain are used as controls (Control-1 for BS3610 and Control-2 for BS168). Right-panel shows fluorescence intensities of reporter strains after 24 h incubation. Scale bars are 100 μm.

The strategy for multiplexing two reporter strains for a differential antimicrobial screening was next demonstrated for complex environmental microbiological samples. We screened for anti-bacterial active compound producers from three different soil sources, (1) pristine soil (SB, the Schwellenburg, Thuringia natural conservation area), (2) garden soil (GS, rich in nutrients), and (3) tar-contaminated soil (TC, highly exposed to oil contaminations). We applied our established protocol for representative extraction of microbial cells from the soil samples to collect microbes for droplet cultivation^14^. Droplets with soil microorganisms at single cell inoculation were generated with medium comprising soy-mannitol medium and a soil nutrient extract (see methods section) and incubated for 3–4 weeks. Growth of diverse microorganisms with different sizes and morphologies were observed (Figs. 4a and S13). A mixture of reporter strains (20:80 ratio of *E. coli* EC081 and *B. subtilis* BS168) was picoinjected to droplets from SB soil sample. Additionally, a second strain of *B. subtilis* BS3610, producing red fluorescence mKate, was also tested and used for picoinjecting (at 20:80 ratio of EC081 and BS3610) to droplets from GS and TC soil samples. This *Bacillus* strain (BS3610) showed homogenous growth and improved red fluorescence in the microtiter plate assay (Fig. S3). After picoinjection and ~ 24 h incubation, fluorescence signals were analysed (Figs. 4b and S14). As most of the droplets were either initially empty (due to Poisson distribution at λ = 0.4 during encapsulation of soil-derived microorganisms) or had limited growth of soil organisms, most droplets had both high green and high red fluorescence intensities showing of lack of inhibition of our reporters.

Droplets with inhibition of either one or both reporter strains were observed in all three soil samples and were targeted for screening (Figs. 4b, S15). In all sorting campaigns, sorted droplets were deposited onto nutrient broth agar (NBA) medium in Petri dishes, which were incubated for recovery of strains and further analysis. NBA medium was used for increasing the chance for growth of many bacteria from the soil sample (unlike in the above section with the model-library screening, which targeted only *Streptomyces*). However, several colonies of reporter strains were also observed (confirmed by green or red fluorescence under UV light) (Fig. S16). As discussed previously, this might be due to favourable growth conditions for reporter strains on agar medium, especially in comparison to slow growing environmental strains. Nevertheless, some non-fluorescent colonies were re-streaked to obtain pure colonies. While some colonies were lost during this process due to cultivation complexities^13^, some cultivated colonies with unique morphologies were selected for further validation analysis against our reporter strains in microtiter well plates.

From the pristine soil from natural reserve area (SB) sample, droplets with both low red and green fluorescence were targeted for sorting (Fig. S15). All isolated colonies (labelled as ‘SB-x’) inhibited growth of both reporter strains (Fig. 4c; Table 1) in the microtiter plate validation experiment. From garden soil (GS), droplets with high red and low green fluorescence intensities (selectively inhibiting growth of *E. coli*) were screened, targeting anti-Gram-negative active compound producing microorganisms. Two isolated strains (labelled as ‘GS-x’) were found to have specific activity against Gram-negatives (Fig. 4c; Table 1). Similarly, anti-Gram-negative or broad-spectrum activity were targeted from the tar-contaminated (TC) soil sample in two rounds of screening. Out of 3 selected isolates (labelled as ‘TC-x-x’), only one isolate showed the expected inhibition profile. The other two isolates either inhibited one or both reporter strains. In total of 13 isolates, 9 showed the predicted and pre-selected activity, demonstrating ~ 69% specificity success rate. It is to be noted that only one isolate did not show any confirmed activity, while 12 isolates were bioactive (~ 92% activity rate). These initial validation experiments were performed equivalent to the droplet experiments, with the reporter strains being added to pre-grown whole cell cultures of the isolates. Further analysis with supernatants of isolates showed similar but sometimes more limited inhibition profiles (i.e. not the full spectrum of the selected inhibition profile could be confirmed, highlighted with bolditalics in Table 1 and Fig. S17), with only 6 isolates (~ 46%) resulting fully in the predicted activity. This could be due to necessity of a close interaction between producer and reporter cells for antimicrobial function, or the nature of the active compounds being not very soluble in the aqueous supernatant phase.

The 16 s rRNA gene region of these strains were amplified and sequenced for identification. The isolated strains were classified into classes Bacilli, Gammaproteobacteria and Actinomycetes. Many isolated strains were known to have broad-spectrum antibacterial activity. A complete list of obtained strains, their inhibition profile in microtiter plate assay, and taxonomic classification based on Sanger sequencing is presented in Table 1.

We then extended the characterization of isolates to standard agar-diffusion bioactivity assays with a diverse microbial pathogen test panel (Supp Table S3) using culture supernatants and solvent extracts. We also included our reporter strains again in this standard assay for reference. Solvent extracts from two isolates (SB-3 and TC-2-1) were confirmed to be active against both reporter strains (Supp Table S3). Remarkedly, those two isolates showed inhibition towards other standard bioactivity test panel microorganisms including *Staphylococcus aureus* and *Mycobacterium vaccae* (Supp Table S3), indicating production of natural products potentially active beyond our targeted reporter strains. One isolate (SB-3, *Serratia marcescens*) was further cultivated in larger volumes in different growth media and the respective organic extracts were analysed by HPLC-HRMS (Supp Info HPLC-HRMS analysis of extracts). Thus, a compound with *m/z* 515.3333 ([M + H]^+^, calcd. for C_26_H_47_N_2_O_8_ *m/z* 515.3327) along with several congeners were detected that correspond to the molecular composition of antibiotic serratamolides (e.g. serratamolide A, Figs. S18-S20), potentially explaining the observed antimicrobial activity of the strain. This finding validates the successful implementation of the screening platform from a complex microbial sample. However, it needs to be confirmed if the detected compound indeed is responsible for the observed antimicrobial activity. This isolate and further isolated strains, inhibiting one or both reporter strains, now serve as resource for the deeper investigation into their antimicrobial properties.

**Table 1:** Classification and activity validation of isolates obtained from the multiplexed screening of different soil samples by Sanger sequencing of 16 s rRNA gene. GenBank accession numbers listed under “GenBank accession no. (this study)” correspond to 16 s rRNA gene sequences generated in the present study. Nearest matched strains and reference accession numbers were identified by BLAST analysis.

Isolates	Nearest matched strain (NCBI)	Class	Identity (%)	Reference accession no.	Targeted activity	Inhibition with cell suspension	Inhibition with supernatant	Known activity/compounds from literature	GenBank accession no. (this study)
SB-3	*Serratia marcescens*	Gammaproteobacteria	99.79	NR_114043.1	Both	**Both**	***Only B. subtilis***	Broad-spectrum (prodigiosin, serrawettin W1)^35,36^	PX845374
SB-13	*Micrococcus luteus*	Actinomycetes	99.44	NR_075062.2	Both	**Both**	**Both**	Broad-spectrum^37^	PX845375
SB-18	*Micrococcus yunnanensis*	Actinomycetes	99.57	NR_116578.1	Both	**Both**	**Both**	Broad-spectrum^38^	PX845376
SB-19	*Micrococcus yunnanensis*	Actinomycetes	99.79	NR_116578.1	Both	**Both**	**Both**	Broad-spectrum^38^	PX845377
SB-27	*Stenotrophomonas pavanii*	Gammaproteobacteria	99.44	NR_118008.1	Both	**Both**	***Only E. coli***		PX845378
SB-28	*Stenotrophomonas pavanii*	Gammaproteobacteria	98.96	NR_118008.1	Both	**Both**	***Only E. coli***		PX845379
GS-2	*Bacillus subtilis*	Bacilli	99.79	NR_102783.2	Anti-*E. coli*	**Only ** ***E. coli***	**Only ** ***E. coli***	Broad-spectrum (surfactins, fengycins)^39,40^	PX845380
GS-7	*Bacillus subtilis*	Bacilli	99.58	NR_102783.2	Anti-*E. coli*	**Only ** ***E. coli***	**Only ** ***E. coli***	Broad-spectrum (surfactins, fengycins)^39,40^	PX845381
GS-8	*Bacillus stercoris*	Bacilli	99.86	NR_181952.1	Anti-*E. coli*	None	None		PX845382
GS-41	*Escherichia fergusonii*	Gammaproteobacteria	99.44	NR_074902.1	Anti-*E. coli*	***Both***	**Only ** ***E. coli***		PX845383
TC-2-1	*Bacillus stercoris*	Bacilli	99.72	NR_181952.1	Anti-*E. coli*	***Both***	***Both***		PX845384
TC-4-1	*Micrococcus yunnanensis*	Actinomycetes	99.15	NR_116578.1	Both	***Only E. coli***	***Only E. coli***	Broad-spectrum^38^	PX845385
TC-4-4	*Micrococcus yunnanensis*	Actinomycetes	99.36	NR_116578.1	Both	**Both**	***Only E. coli***	Broad-spectrum^38^	PX845386

## Discussion

In this work, we have reached two main goals for our droplet-based ultrahigh throughput screening efforts to discover new antibiotics: (1) We have established and demonstrated an optimized strategy for multiplexing two reporter species for detecting producers of antibiotic substances. And (2) we have validated this multiplexed screening with model and environmental samples of natural product producers.

Our strategy combines a modular workflow for the cultivation of microorganisms^14^ and multi-colour fluorescence detection^34^ in microfluidic droplets. The antibiotic assay consists of a whole-cell phenotypic screening similar to the Waksman method^41^. The antimicrobial activities were determined by inhibition of reporter strains equipped with constitutively expressed fluorescent protein. Hence, inhibition resulted in lower fluorescence intensities. As reporter strains, one Gram-positive and one Gram-negative strain were utilized, considering the different molecular targets those two big microbial groups represent. This allowed screening of many active compounds, possibly having inhibitory activity against either Gram-positive or Gram-negative pathogens within one screening round. Furthermore, this enables the rational selection of producers of either broad band or specific bioactive compounds. We can easily change the sorting algorithms to sort droplets inhibiting only one or both of them. As AMR is increasing due to excessive use of broad-spectrum antibiotics, our approach provides options for identifying more narrow-spectrum active compounds.

We have utilized two model reporter strains: *E. coli* expressing GFP and *B. subtilis* expressing mKate proteins. In the future, multiplexing could be expanded by increasing the number of reporter strains expressing distinct fluorescence proteins. Further tests could be performed selectively with ESKAPE strains, addressing the severity posed by life-threatening pathogens^4^. With the advancement in multi-color fluorescence detection^34,42,43^ and efficient picoinjection strategies^44,45^, simultaneous or differential inhibition of various reporter strains could be anticipated. A target-based screening approach is preferred to find certain types of inhibitors against some pathogens with desired modes-of-action. Our multiplexing strategy could be implemented for such type of target-based whole-cell screening approaches like RNA antisense based assays^46,47^. Reporter strains with antisense RNA and control strains could be modified to express two distinct fluorescence proteins and could be utilized for rapid screening focused on selective targets. Similarly, based on antibiotic-triggered mRNA expression profiles^48,49^, multiple promoter-reporter gene fusions can be constructed using highly induced promoter region by antibiotic activity. Also, multiple reporter strains expressing different fluorescent proteins could be utilized for demonstrating interference to major biosynthetic pathways like cell wall biosynthesis, protein biosynthesis, or DNA biosynthesis. This would fine-tune our approach and allow comprehensive target-based high-throughput screening for active natural products.

To prove functionality of our screening approach, we applied the reporter strain assay to undefined microbial resources from different soil environments and several isolates were obtained based on targeted sorting criteria. The targeted selection and sorting efficiency were ~ 86% and ~ 69% for a model library of antibiotic producing *Streptomyces* strains and the natural soil samples, respectively, when comparing the droplet screening outcome towards our reporters with bioactivity tests against the same in microcultivation experiments. However, the targeted hit rate of isolates from the soil samples is reduced during the bioactivity test with supernatants in microtiter plates (only ~ 46%) or with solvent extracts on agar-based tests (either methanol or ethyl acetate extracts at 1:10 ratio solvent to culture supernatant, only two out of 13). The main difference of both approaches is a direct contact of the producer culture broth with the reporter strain versus a diffusion-based exposure of the test strains to the culture supernatant through an agar matrix. Thus, this discrepancy of the two approaches could be due to the necessity of close contacts between producer cells and reporter strains, concentration or solubility issues of active compounds, or nutrient competitions. The observation that most isolates retained inhibitory activity (12 out of 13 isolates) in cell-free supernatant assays suggests that the observed inhibitory effects are predominantly attributable to diffusible antimicrobial metabolites rather than nutrient competition. Also, in case of some anti-microbial proteins, they might not be diffusible on agar, limiting their activity. To counteract the significantly lower effective concentration of antimicrobial metabolites when using direct culture supernatant in the bioactivity assay, we also tried to concentrate the active compounds with methanol and ethyl acetate as solvents. For one of the two strains this approach resulted in a confirmation of antimicrobial activity against Gram-positive bacteria (strain SB-3). As a strategy forward to increase the success rate of the droplet screening campaign, we propose to develop a new droplet microfluidics workflow to increase the initial selection of hits and collect a bigger pool of droplets of interest (~ 1000 or more) for detailed testing on an automated (robotic) liquid handling platform to increase chances of success. We are working towards establishment of such a workflow for handling larger numbers of sorted droplets and combining it with mode-of-action-based reporter strain assays on a microtiter plate robotic platform.

To minimize the redundant findings of previously identified compounds, reporter strains with resistance genes for common antibiotics could be implemented. A dereplication step could be implemented by coupling mass-spectrometry^50,51^ or IR-based^52^ detection in the current microfluidic workflow. Besides a deeper early analysis of potential droplets of interest, sampling from exotic habitats or niches like deep sea-water, insect, human, or animal gut^23,25,27,53^ could result in a higher chance of success. It is also noteworthy to consider microbial interactions in nature, living in community with two or more species and supporting growth^54^ or acting as elicitors for producing secondary metabolites^55,56^. As our current workflow targets single cells, we are unable to capture those natural product producers. However, cell concentrations could be effectively diluted during droplet generation to establish micro-communities inside of droplets, with a probability of random pairing or grouping of ecologically relevant partner species.

Several factors still remain challenging with the current droplet-based screening platform. One key aspect is inter-droplet transport of small molecules. As droplets are surfactant-stabilized boundary, rather than a mechanical barrier, small hydrophobic molecules with relatively high partition coefficients can leak from droplets into the oil^57,58^, and minimizing the potential inhibitory activity of a producer droplet. In contrast, the chance of false positive detection is very low as oil is constantly re-circulated during incubation and leaking compounds will get diluted to very low concentration. We expect ongoing developments of new surfactant formulations (like dendronized fluorosurfactant or pickering emulsions)^59,60^, using thermosetting oil to create a cured oil matrix around the droplets^61^, decreasing surfactant concentrations or using additives (like BSA or cyclodextrin)^62^ could minimize this effect.

Lastly, the process for isolating strains after droplet deposition still needs further optimization to minimize the frequent (co)-isolation of reporter strains from agar plates. Currently, reporter strains, which were either partially inhibited or static inside of droplets, are able to grow faster on agar medium than environmental isolates. For simplifying isolation and analysis of putative antibiotic producers, it would be ideal to have reporter strains with kill switch mechanisms based on metabolic auxotrophy^63,64^ for essential amino acids or synthetic auxotrophy^65,66^ that creates a dependency on non-natural amino acids for survival. This would allow changing growth media or removing some nutrient constituents on agar plates for selectively inhibiting all reporter cells. Another, more complicated approach would be to modify the gene circuits of the reporter strains to induce autotoxin production or to repress the expression of essential genes under the desired biocontainment conditions^67^. Furthermore, reporter strains with temperature-sensitive kill switch mechanism^68^ could also be developed and implemented in a screening approach.

## Conclusion

Our screening approach possesses the capability for screening and differentiating possible antibiotic producers inhibiting either Gram-positive or Gram-negative microbes exclusively or simultaneously. It combines the ultrahigh-throughput capacity of droplet microfluidics and phenotypic screening using multiple reporter strains, creating an effective platform for multiplexed antibiotic screening and even beyond, a promising tool for exploring active compounds in diverse microbial communities.

## Materials and methods

### Microorganisms and culture conditions

*Escherichia coli* EC081 (pMK3c2GFP), constitutively producing GFP, *Bacillus subtilis* BS168 (amyE::hy-mKATE::Cm), and *B. subtilis* BS3610 (amy::hy-mKATE: Spec) constitutively producing mKate^69^ were used as reporter strains. Reporter strains were cultured for 16 h in LB media with 100 µg/ml kanamycin (for *E. coli* EC081), 10 µg/ml chloramphenicol (for *B. subtilis* BS168) or 100 µg/ml spectinomycin (for *B. subtilis* BS3610) at 37 °C and 200 rpm. From the cultures, pre-cultures were inoculated into the medium without antibiotics to an optical density OD_600_ of 0.1 and cultivated until the mid-exponential phase, corresponding to an OD_600_ of about 2. Then, cells were pelleted and re-suspended in 2.5X LB medium. For simultaneous picoinjection of both reporter strains, *E. coli* and *B. subtilis* were mixed in a ratio of 20:80 to obtain a final OD_600_ of 2 (equivalent to ~ 1.6e9 cells/mL). For the optimization of this inoculum ratio, the two reporter strains were co-cultivated in microtiter plates in the microbioreactor system BioLector II (m2p-labs, Germany) at different inoculation ratios to obtain a balanced co-culture composition despite differing growth rates.

The Actinomycetales strains used as antibiotic producers in this study are listed in Table S2. They were cultured in MMM medium (Table S1). For cultivation in droplets, spore suspensions of the strains were diluted in medium to a final concentration of 5 × 10^7^ spores/mL, and droplets were generated from the diluted suspension at a frequency of higher than 1 kHz resulting in ~ 10 spores/droplet. Generated droplets were incubated with the dynamic droplet incubation setup^33^ placed in a humid chamber at 28 °C.

For the model library experiment, a bacterial community was generated by mixing known *Streptomyces* strains (Table S2). Spores of five selected strains were mixed. The mixed spore suspension was encapsulated in droplets (at λ of 0.4 spores/droplet) and incubated for 3 days before picoinjection of the reporter strains.

### Soil sampling and extraction

For screening antibiotic producers from environmental habitats, three soil samples were collected from different locations; a natural reserve area (“Schwellenburg” close to Erfurt, Germany, marked as ‘SB’), garden soil (Jena, Germany, marked as ‘GS’) or tar-contaminated soil (“Lützkendorf” near Halle, Germany, marked as ‘TC’). Droplets were generated from soil sample extracts following a previously reported protocol^14^. In brief, soil was mixed with water to prepare slurry and was agitated for 2 h. After sedimentation, supernatant with cells were filtered-through a cell strainer (40 μm), aliquoted, and stored at −20 °C until use. Also, a portion of supernatant was centrifuged at 15,970 g for 20 min and sterile filtered using a 0.2 μm membrane filter. This was used as cold-extracted soil extract (CESC, to supplement nutrients from original soil sample). For droplet generation, extracted cells were mixed with 12% (v/v) supernatant of soy mannitol medium, 50% (v/v) CESC, and 38% distilled water. Generated droplets were incubated in a dynamic droplet incubator at 20 °C.

### Fabrication of microfluidic chips

All microfluidic structures were designed in CAD software (Supp Figs. S24-S26) and fabricated with soft-lithography protocols by using laser-printed glass molds as described previously^70^. Briefly, 3D-channel structures, including optical parts and electrodes, were initially designed in AutoCAD 2015 (Autodesk Corp, USA). Glass molds were fabricated on fused silica slides using femtosecond laser machining technology (FEMTOprint SA, Switzerland). Common soft-lithography protocols were used to obtain intermediate molds and working chips in polydimethylsiloxane (PDMS, Sylgard 184, Dow Corning, Germany). The standard PDMS mixture was poured onto the mold, degassed, and thermally polymerized at 70 °C for 3 h. Polymerized PDMS was peeled off from the mold, cut into individual chips, and holes were punched for fluidic connections using a biopsy punch with a plunger (Ted Pella, USA). The resulting PDMS replica chips for droplet generation were plasma bonded to microscope glass slides, and those for picoinjection and sorting to indium tin oxide coated glass slides (Delta Technologies, USA). Channels in PDMS chips were flushed with 1% (vol/vol) trichloro(1*H*,1*H*,2*H*,2*H*-perfluorooctyl)silane (Sigma) in Novec HFE 7500 (3 M, Germany). Electrodes in chips were generated by filling low melting solder (Indalloy 19, Indium Corporation of America, USA) into allocated electrode channels while the chip is being heated to 100 °C.

### Fluidic assembly and chip operation

Liquids in microfluidic chips were actuated by high precision syringe pumps (neMESYS, CETONI GmbH, Germany) and pressure pumps (MFCSTM-EZ, Fluigent, France). Polytetrafluoroethylene (PTFE) tubings (0.5 mm ID, SCP GmbH, Germany) were inserted into the inlets and outlets of the microfluidic chips for transferring fluids. Microfluidic operations were monitored with a high-speed camera (Pike F-032B camera, Allied Vision Technologies, Germany) in an inverted microscope (Axio Oberver Z1, Carl Zeiss, Germany). Novec (HFE 7500, 3 M) oil with 0.5% fluorinated surfactant (Picosurf, Sphere Bio) was used as a continuous phase. The characteristic pressures applied for droplet generation were 500 and 350 mbar for the oil and aqueous phases respectively, for a chip with a flow-focusing junction of 50 μm height and 50 μm width, resulting in a droplet generation frequency of 1.5 kHz.

### Optical setup and configuration

A single-mode fiber (LASOS Lasertechnik, Germany) with a cladding diameter of 125 μm, a core diameter of 6 μm and a numerical aperture (NA) of 0.14 was used as an incident fiber. Multi-mode fibers (Thorlabs, Germany) with a cladding diameter of 125 μm, a core diameter of 50 μm and NA of 0.22 were used as detection fibers. The incident fiber was coupled to a beam combiner containing lasers with different wavelengths (405 nm, 488 nm, 561 nm, and 639 nm). The fiber for detection of scattered light was coupled to a photomultiplier module (PMT; H10723-20, Hamamatsu Photonics, UK). Similarly, the fiber for detection of fluorescence was coupled to a PMT (H10721-20, Hamamatsu Photonics, UK) with a quad-bandpass filter (440/521/607/700 HC Quadband Filter, AHF, Germany).

### Visualization of droplets

Microfluidic droplets with encapsulated spores or bacterial cells were loaded into an observation chamber and visualized with a PCO.edge 5.5 m camera (PCO, Germany). Different fluorescent channels were imaged using excitation light from a SpectraX light engine (Lumencor, Germany) with standard red and green filters (Filter sets 43 HE and 38 HE, Carl Zeiss, Germany).

### Image analysis

Images were analyzed for identifying droplets and determining grey values in each droplet by using the image analysis software Fiji^71^. The bright-field images were binarized for detecting the droplet borders. For binarization, the local threshold method default (radius 10 pixels) was used. Black foreground structures were dilated in a lateral direction by 5 pixels and subsequently inverted. The “Analyze Particles” function was used to detect circular shapes with the radius of droplets (2000–20000 pixel) and defining regions of interest (ROIs). Radiuses of ROIs were reduced by 5 pixels to remove interference from droplet borders during measurements. ROIs were used in all images to determine the average grey value for the respective fluorescence channels. Measured grey values were analyzed and visualized in R^72^.

### Picoinjection

Droplets with fully grown cultures of bacteria were reinjected into a optimized picoinjection chip (Supp Fig. S25) along with an aqueous phase of reporter strains (Supp Info Picoinjection process and Fig. S21). Droplets of reporter strains were fused and added to by-passing- droplets (Supp Video S1) by applying an alternating electrical field through an integrated electrode using a function generator (AFG-2005, GW Instek, China) and a high voltage amplifier (model 2210-CE, Trek, USA). The function generator was operated in sine-wave mode at 200 mV (1000x amplified), 20 kHz and duty cycles of 50%. Picoinjected droplets were collected and incubated for 24 h at 37 °C. The characteristic flow rates were 140 nL/s and 50 nL/s for spacing oil and droplets respectively. The pressure of 120 mbar was applied for aqueous reporter strains, resulting in a picoinjection frequency of ~ 300 droplets/s.

### Sorting and signal analysis

For sorting, droplets were reinjected into a sorting chip (Supp Fig. S26) with integrated capillary tubing. The characteristic flow rates were 150 nL/s, 30 nL/s, and 75 nL/s for first-spacing oil, droplets and second-spacing oil respectively. Additionally, a positive pressure of ~ 30 mbar was applied to the positive channel outlet, to retain droplets into the collection chamber. The scattered light was collected by an optical fiber and detected by a PMT (Supp Fig. S10). Similarly, emitted fluorescence light was collected by a detection fiber and guided to a PMT. Multiple fluorescence signals were simultaneously detected by a single PMT using frequency division multiplexing^34^. The signal from the PMT was pre-amplified using a trans-impedance amplifier and connected to a lock-in amplifier (HF2LI, Zurich Instruments, Switzerland). Excitation lasers with different wavelengths were modulated in specified frequencies. These frequencies were used by a lock-in amplifier to demodulate and filter signals into individual modulation frequencies. The demodulated output signals from the lock-in amplifier were collected, recorded, and visualized using a DAQ card (USB-1608GX, Measurement Computing) with a custom-written LABVIEW^®^ program. Recorded data were further processed and analyzed using R script.

### Electronics for droplet sorting

For sorting droplets, either scattered signals or blue fluorescence signals (background fluorescence due to media components) were used as droplet markers (Supp Info Sorting setup and electronics). Red and green fluorescence signals were used to detect the inhibition of the reporter strains. The scattered light signal from the PMT was fed into a trigger box (Supp Fig. S22). Whenever a droplet passes through the optical interrogation region, a digital trigger signal was created. The demodulated fluorescence signals from the lock-in amplifier were fed into two trigger boxes (one for red and another for green). Threshold values for fluorescent wavelengths were predefined. Droplet signals exceeding the predefined threshold values generated digital trigger signals for each wavelength. The digital signals from all three trigger boxes were fed into an additional Arduino chip (Supp Fig. S22). Whenever a droplet marker signal was detected as high, signals in fluorescent channels were analyzed. Depending on the gating setup, an output pin was set high to trigger a function generator (Arduino code in Supp Info). In the Arduino, an initial delay of few milliseconds (8 milliseconds in our case) is applied to match the travel time of droplets from detection zone to the sorting region. After this delay, a trigger-OUT signal for the high-voltage electrode is generated (3 milliseconds in our case) to direct the droplet into the positive channel. The function generator was operated in a burst mode with 450 mV (1000x amplified), 50 cycles, 10 kHz and 50% duty cycle. This setup enabled proper adjustment of all critical sorting parameters. With this configuration, and considering the clock speed of the Arduino Uno (16 MHz), we were able to sort droplets at a frequency ~ 150 droplets/s without any technical limitations, enabling ultra-high throughput sorting. Droplets fulfilling the gating requirements were actively sorted into a positive channel and directed into a droplet collection structure on the chip (Supp Video S2).

### Isolation and recovery of microorganisms from sorted droplets

A multi-layer 3D chamber structure connected to a sorting structure was designed and fabricated, as previously described^73^. In brief, an oval structure resembling a football stadium was designed with droplet inlet on one end and narrow exit channels surrounding the structure (Supp Info Recovery of droplets). The narrow exit channels, 15 μm wide and 50 μm deep, were used as an exit for removing excess oil while keeping the sorted droplets trapped in the chamber (Supp Fig. S23). A secondary exit channel was fabricated for retrieving trapped droplets at the end of the experiment. A pressure pump controlled the exit channel of the collection chamber. Once the collection chamber was fully occupied, or the sorting process was finished, a back pressure was applied from the exit channel and droplets were recovered through capillary tubing onto an agar plate (Supp Video S3). The capillary tubing was connected to the XYZ-positioning system and programmed for spiral movement on top of the agar plate (Supp Fig. S23). The growth of microorganisms was determined by counting colonies after incubation.

### Sanger sequencing of isolates

Colonies arising from droplets were initially controlled for presence of reporter strains by taking images of agar plates under UV light. Non-fluorescent colonies were randomly selected and re-streaked. For Sanger sequencing, the 16 s rRNA gene was amplified from colonies using primers 27 F (AGAGTTTGATCMTGGCTCAG) and 1492R (CGGTTACCTTGTTACGACTT) and Hotstart Green PCR Kit (Thermo). The PCR included the following steps: pre-denaturation (95 °C, 5 min), 25 cycles of denaturation (95 °C, 30 s), annealing (49 °C, 30 s), elongation (60 °C, 1 min), and final elongation (72 °C, 10 min). After gel electrophoresis, expected bands at ~ 1400 bp were purified using a gel extraction kit (Monarch) and sequenced (Eurofins Genomics) using both primers as above. The forward and reverse sequences (SI Data Table) were used to obtain consensus sequence (using pairwise alignment from Biostrings package in R script) resulting in a nearly full-length 16 s rDNA gene. The obtained sequences were blasted in the NCBI database (https://blast.ncbi.nlm.nih.gov/Blast.cgi) and the highest-matching strains were identified.

### Microtiter well plate and agar diffusion assay

For the microtiter well plate assay, isolated environmental strains were cultivated in shake flasks for 10 days at 28 °C, 160 rpm. Isolate supernatants were obtained by centrifugation at 15,000 xg for 15 min. Culture suspension and supernatants were stored at −20 °C until testing. Reporter strains (*E. coli* EC081, *B. subtilis* BS168 or *B. subtilis* BS3610 cultivated in LB media) was added to culture suspension or supernatants and incubated in the microbioreactor system BioLector II (m2p-labs, Germany) at 37 °C and 600 rpm. Scattered signal (as Biomass measurement) and fluorescence signals (in GFP and mCherry channels) were continuously monitored during incubation.

Agar diffusion assay was performed as described previously^13^. A panel of test strains, which included different species of Gram-positive and Gram-negative bacteria as well as fungi was used (see Table S3). The antibiotics ciprofloxacin 5 µg/ml and amphothericin B 10 µg/ml were used as reference controls for bacterial and fungal test strains, respectively.

### Data analysis and visualization

GraphPad Prism and R/RStudio were used to make plots and perform statistical analyses.

## Supplementary Information

Below is the link to the electronic supplementary material.

Supplementary Material 1

Supplementary Material 2

Supplementary Material 3

Supplementary Material 4

Supplementary Material 5

## Data Availability

The datasets generated and analysed in the current study are available from the corresponding author on reasonable request. 16 S rDNA sequence data of all isolates was deposited in GenBank under accession number PX845374-PX845386.
