# Supplementary material for: Multiplexing antibiotic screening assay in droplet microfluidics
Source: Sci Rep. 2026 Jun 3;16:17102. doi: 10.1038/s41598-026-55537-2 (PMC13230938; doi:10.1038/s41598-026-55537-2)
Supplement: Supplementary file 1 — Supplementary Material 1 [file 41598_2026_55537_MOESM1_ESM.docx]

**Supplementary Information**

**Multiplexing antibiotic screening assay in droplet microfluidics**

Sundar Hengoju^a,b^, Karin Martin^a^, Kirstin Scherlach^b^, Martin Roth^a^, and Miriam A. Rosenbaum^a,c,^*

^a^ Bio Pilot Plant, Leibniz Institute for Natural Product Research and Infection Biology – Hans-Knöll-Institute, 07747 Jena, Germany

^b^ Biomolecular Chemistry, Leibniz Institute for Natural Product Research and Infection Biology – Hans-Knöll-Institute, 07747 Jena, Germany

^c^ Faculty of Biological Sciences, Friedrich Schiller University, 07743 Jena, Germany

* Correspondence: Miriam.rosenbaum@leibniz-hki.de

Contents

[Media composition 2](#_Toc214538967)

[Co-cultivation of reporter strains 2](#_Toc214538968)

[Inhibition of reporter strains by *Streptomyces* 4](#_Toc214538969)

[Model library of *Streptomyces* 5](#_Toc214538970)

[Selection of agar media for growth of *Streptomyces* 8](#_Toc214538971)

[Validation of sorted droplets from model library 9](#_Toc214538972)

[Droplet screening of environmental soil 10](#_Toc214538973)

[Validation of isolated colonies from droplet screening of environmental soil 14](#_Toc214538974)

[HPLC-HRMS analysis of extracts 16](#_Toc214538975)

[Supplementary technical methods 20](#_Toc214538976)

[Picoinjection process 20](#_Toc214538977)

[Sorting setup and electronics 21](#_Toc214538978)

[Recovery of droplets 23](#_Toc214538979)

[References 25](#_Toc214538980)

# Media composition

Table S1. List of media compositions

| **Medium** | **Composition** | **Use** |
| --- | --- | --- |
| MMM | 2 g/L yeast extract (BD Bioscience, Belgium) + 2 g/L beef extract + 15 g/L malt extract (BD Bioscience, Belgium) | Cultivation of *Streptomyces* for model library screening |
| LB | 10 g/L Bacto tryptone (BD Bioscience, Belgium) + 5 g/L Yeast extract (BD Bioscience, Belgium) + 10 g/L NaCl (Merck, Germany) | Cultivation of reporter strains |
| MM | 1.982 g/L (NH_4_)_2_SO_4_ + 2.423 g/L Tris/HCl + 0.993 g/L NaCl (Merck, Germany) + 0.426 g/L K_2_SO_4_ (Roth, Germany) + 0.197 g/L MgSO_4_ x 7 H_2_O (Roth, Germany) + 0.0777 g/L CaCl_2_ (Roth, Germany) + 10 ml trace element solution (1.39 g/L FeSO_4_ x 7 H_2_O + 0.6814 g/L ZnCl_2_ + 0.989 g/L MnCl_2_ x 4 H_2_O + 1.1897 g/L CoCl_2_ x 6 H_2_O + 6.1793 g/L NH_4_MoO_24_ x 4 H_2_O) + 9 g/L L-asparagine (Sigma-Aldrich, Germany) + 0.340 g/L KH_2_PO_4_ (Merck, Germany) | Agar plates for recovery of model library isolates |
| MM+Glucose | MM + 9 g/L glucose (VWR International, USA) |  |
| MM+Ribose | MM + 10 g/L ribose (Sigma-Aldrich, Germany) |  |
| MM+Xylose | MM + 10 g/L xylose (Sigma-Aldrich, Germany) |  |
| MM+No Glu | MM + 0 g/L glucose (VWR International, USA) |  |
| MM+Glu+NA | MM + 10 g/L glucose (VWR International, USA) + Nalidixic acid (Sigma-Aldrich, Germany) |  |
| NBA | 2 g/L yeast extract (BD Bioscience, Belgium) + 1 g/L beef extract + 5 g/L bacto peptone (BD Bioscience, Belgium) + 5 g/L NaCl (Merck, Germany) + 15 g/L agar (Roth, Germany) | Agar plates for recovery of soil screening isolates |
| SM | 20 g/L soy coarse flour (Schkade Landhandel, Germany) + 20 g/L mannitol (Merck, Germany) | Cultivation of isolates in shake flasks |

# Co-cultivation of reporter strains

We initially tested two reporter strain, *E. coli* EC081 expressing GFP and *B. subtilis* BS168 expressing mKate proteins. Cocultivation of these two reporter strains were tested in droplet (Fig. S1) and microtiter well plates (Fig. S2) using LB media.


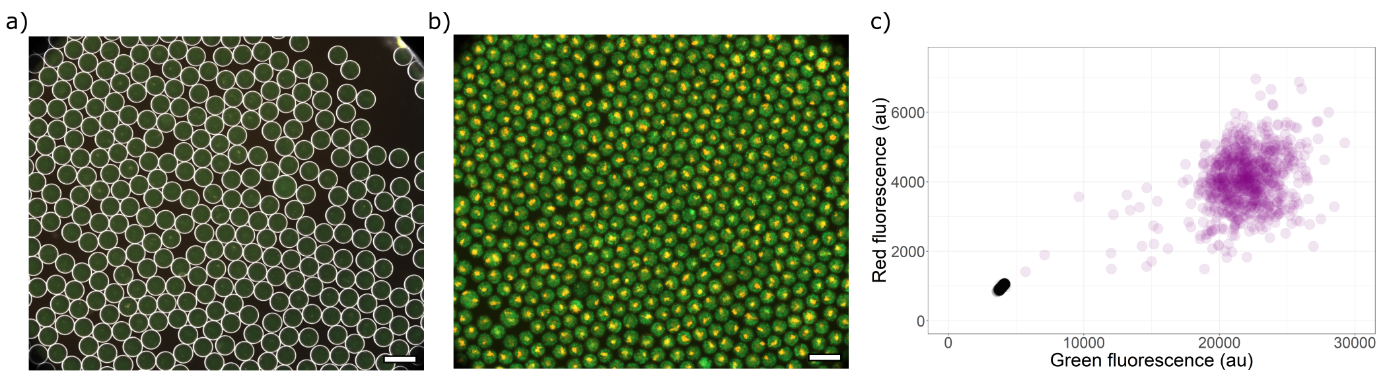


Fig. S1. Growth of two reporter strains (*E. coli* EC081 and *B. subtilis* BS168) in droplets. Droplets were generated with a mixture of both reporter strains at 1:1 ratio with inoculation OD of 0.2, assuring all droplets are filled. Generated droplets were incubated at 37 °C for overnight using dynamic droplet incubator. Images were taken before (a) and after (b) incubation and fluorescence intensities were analyzed. Increase in both green and red fluorescence intensities are observed after incubation (c). Each dot in the scatter plot represents a droplet. N is more than 350 for both conditions. Scale bar is 100 µm.


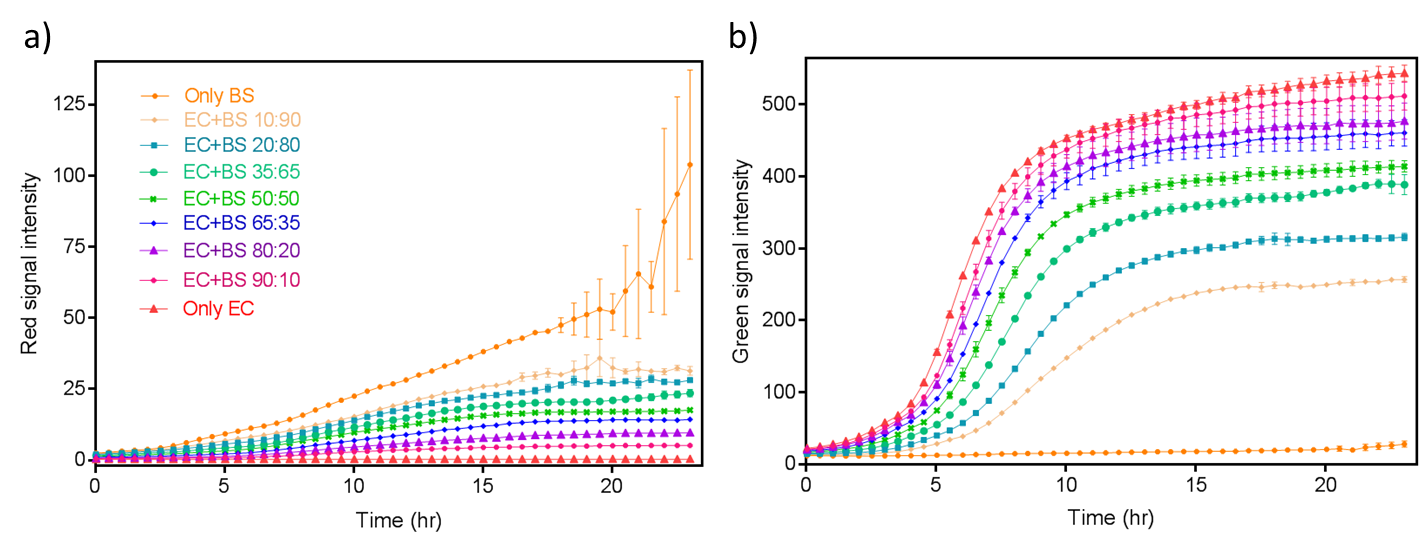
Fig. S2. Co-cultivation of the reporter strains *E. coli* EC081(EC) and *B. subtilis* BS168 (BS) at different inoculum ratios in well plates. Cells were cultivated in LB media at 28 °C and 600 rpm in microbioreactor Biolector device. The growth of the reporter strains was determined by measuring (a) red (for *B. subtilis*) and (b) green (for *E. coli*) fluorescence intensities.

We further tested a second strain of *B. subtilis* BS3610, also expressing mKate. This strain showed slightly improved red fluorescence in the microtiter plate assay, while strain BS168 had larger variability in red fluorescence, which could be due to formation of cell clumps (Fig. S3). The *B. subtilis* strain BS3610 was used in combination with *E. coli* EC081 as reporter strains for screening of garden and tar-contaminated soil samples.


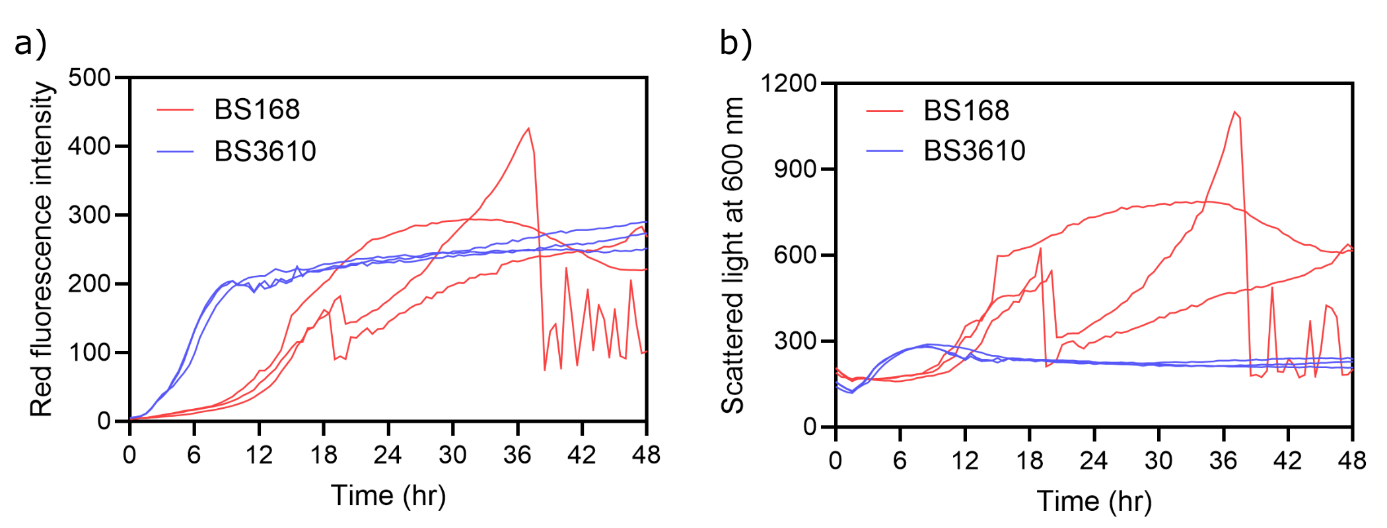


Fig. S3. Comparison of red fluorescence and growth (as scattered light biomass signal) of two mKate producing *B. subtilis* strains, BS168 and BS3610. Three replicates of each strains were cultured in LB media in a microtiter well plate and signals were measured in Biolector.

# Inhibition of reporter strains by *Streptomyces*

Initially, we performed inhibition assays of mixed reporter strains (20:80 ratio of *E. coli* and *B. subtilis*) with supernatants of *S.* *noursei* (SM2-2)*, S. hygroscopicus* (HKI16) *and S. collinus* (43780) to test inhibition profiles in microtiter well plate. *Streptomyces* were cultivated for 4 days in MMM media at 28 °C and supernatants were obtained by centrifugation and sterile filtration using 0.2 um filter. A mixture of the reporter cells in fresh LB medium was added to the *Streptomyces* supernatant in a ratio of 1:1 and incubated for 24 h in a Biolector. Inhibition levels were determined by comparing fluorescence intensities with control samples. We found that supernatant of *S. noursei* inhibited both reporter strains, characteristic for the produced antibiotic nourseothricin^1^, while *S. hygroscopicus* and *S. collinus* inhibited only a single strain, *B. subtilis* or *E. coli,* respectively (Fig. S4).


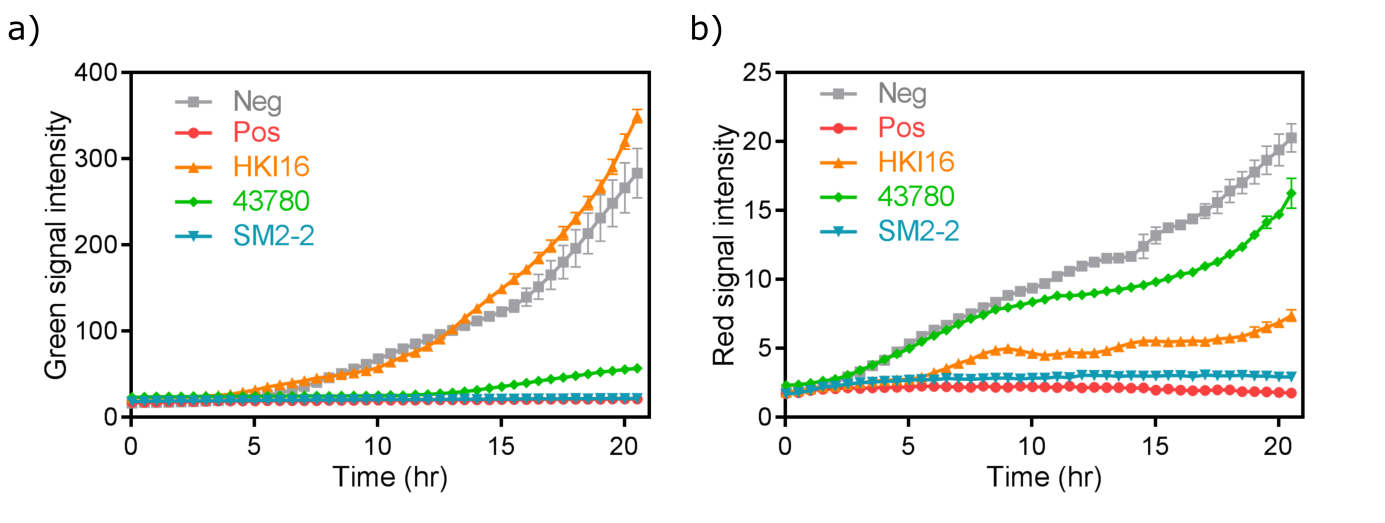


Fig. S4. Inhibition of reporter strains by supernatants of *Streptomyces* cultures. The growth of the reporter strains was monitored by measuring a) green (*E. coli*) and b) red (*B. subtilis*) fluorescence. Supernatants from three *Streptomyces* strains (*S. noursei* SM2-2*, S. hygroscopicus* HKI16 and *S. collinus* 43780) were added to a mixture of the reporter strains (20:80 of *E. coli* EC081 and *B. subtilis* BS168) and cultivated in a Biolector system. Medium (no inhibition) and medium+tetracycline (10 µg/mL, full inhibition)) were used as negative and positive controls, respectively.

For confirming active compound production in droplets, we encapsulated spores of two *Streptomyces* (*S. noursei* SM2-2 and *S. hygroscopicus* HKI16) in droplets along with MMM media at λ ~10 spores/droplet. A third droplet population was also generated using only MMM media. These 3 droplet populations were mixed in equal ratios and incubated for 4 days. Additionally, all three individual droplet populations were also incubated in three separate incubation chambers as control droplets. After incubation we could see dense mycelial growth inside of droplets (Fig. S5). A 20:80 mixture of EC081 and BS168 reporter strains was picoinjected to all droplet populations and further incubated for 24 hrs. Fluorescence images in green and red channels were obtained and analyzed. From image analysis, mixed population grouped into three clouds, which overlap with signal intensities of three control populations (Fig. S6).


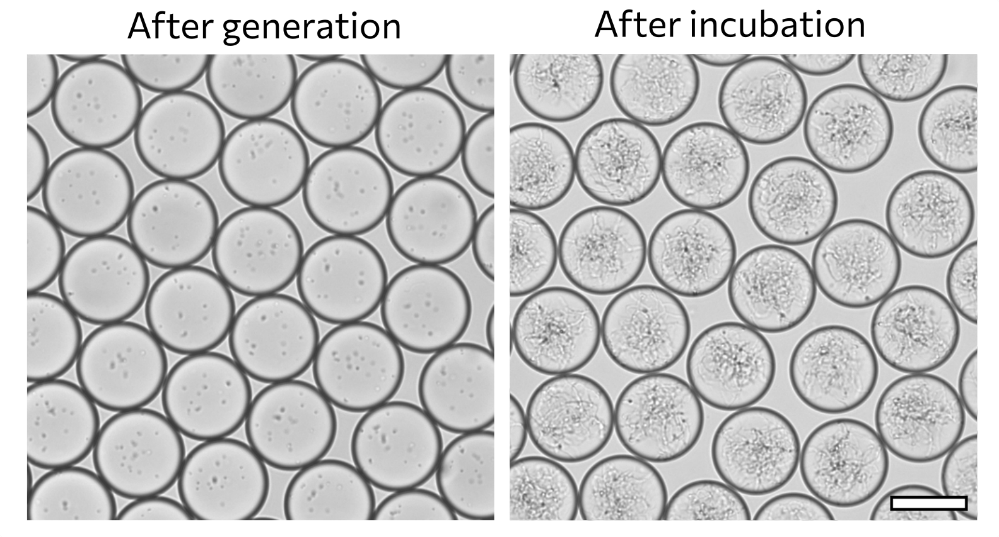


Fig. S5. Representative image showing growth of *Streptomyces (S. hygroscopicus)* in droplets. Droplets were generated with spores of *Streptomyces* at λ ~10 spores/droplet. Dense mycelia are observed after incubation in droplets. Scale bar is 50 µm.


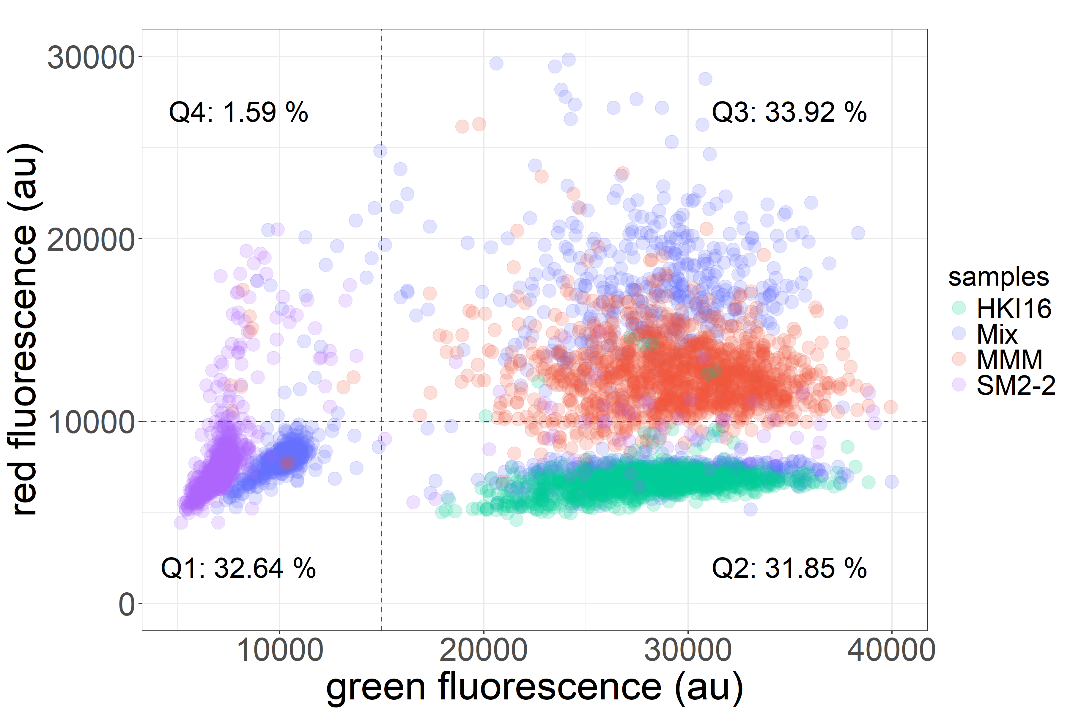


Fig. S6. Classification of a model mixed droplet population by image analysis. Droplets of *S. noursei* (SM2-2), *S. hygroscopicus* (HKI16)*,* and only medium (MMM) were combined to generate a mix population (Mix). Simultaneously, all three droplet populations were incubated separately as controls. After picoinjection of reporter strains (20:80 mixture of EC081 and BS168) to all four droplet populations (mix and three individual controls) and 24 h incubation, fluorescence intensities were determined by image analysis. The mix population (blue dots) was classified into three clusters based on the reporter strain signal and overlapped with the control population signals (purple, green and orange dots). Each dot represents the signal of one droplet.

# Model library of *Streptomyces*

A model library of *Streptomyces* was created by mixing spores of 5 different strains, as listed in the table S2. Some of these strains were tested for their activity against reporter strains on agar plates. For this, strains were cultivated individually in shake flasks in MMM media for 3 days. Using supernatants of these strains, inhibition of reporter strains (EC081 and BS168) was tested using agar-diffusion assay (Fig. S7).

For the droplet screening experiment, the spore mixture of 5 strains was used to generate droplets targeting a single spore per droplet (with λ = 0.4). Generated droplets were incubated, picoinjected with a mixture of reporter strains (20:80 ratio of EC081 and BS168) and used for sorting experiments (Fig. S8). Sorted droplets were dispensed on minimal media (MM) agar plates supplemented with ribose sugar for further colony recovery and isolation (Fig. S9).

Table S2. List of *Streptomyces* strains used in this study (for creating model library) and corresponding identification numbers from Jena Microbial Resource collection (JMRC).

| **Strains** | **Label** | **Antibiotic produced** | **Activity spectrum** | **Reference** |
| --- | --- | --- | --- | --- |
| *S. noursei* JA03890 | SM2-2 | nourseothricin | broad-spectrum | Gräfe *et al.*^1^ |
| *S. hygroscopicus* HKI0016 | HKI16 | staurosporin | anti-Gram positive | Schroeder *et al.*^2^ |
| *S. collinus* IMET43780 | 43780 | kirromycin | anti-Gram negative | Wolf *et al.*^3^ |
| *S. griseus* IMET40235 | HP | streptomycin | snti-Gram positive | Schatz *et al.*^4^ |
| *K. azatica* HKI0216 | HKI216 | alazopeptin | snti-Gram negative | Hata *et al.*^5^ |


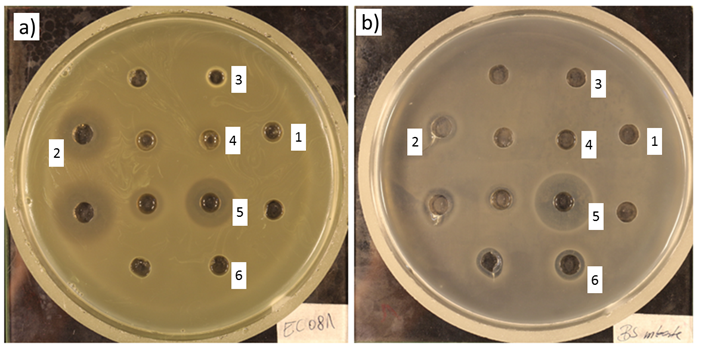


Fig. S7. Inhibition of reporter strains by supernatant of *Streptomyces* cultures. *Streptomyces* strains were grown in MMM medium for 3 days allowing production of active compounds. Supernatants of cultures were pipetted into wells in agar plates which were pre-lawned with reporter strains; a) *E. coli* EC081 and b) *B. subtilis* BS168. 1: Negative control (medium only), 2: Positive control (medium+tetracycline), 3: *S. hygroscopicus*, 4: *S. collinus*, 5: *S. noursei,* and 6: *S. griseus*. Agar plates were incubated for 24 h. Inhibition zones were determined by measuring the diameter of the clear ring around the well.


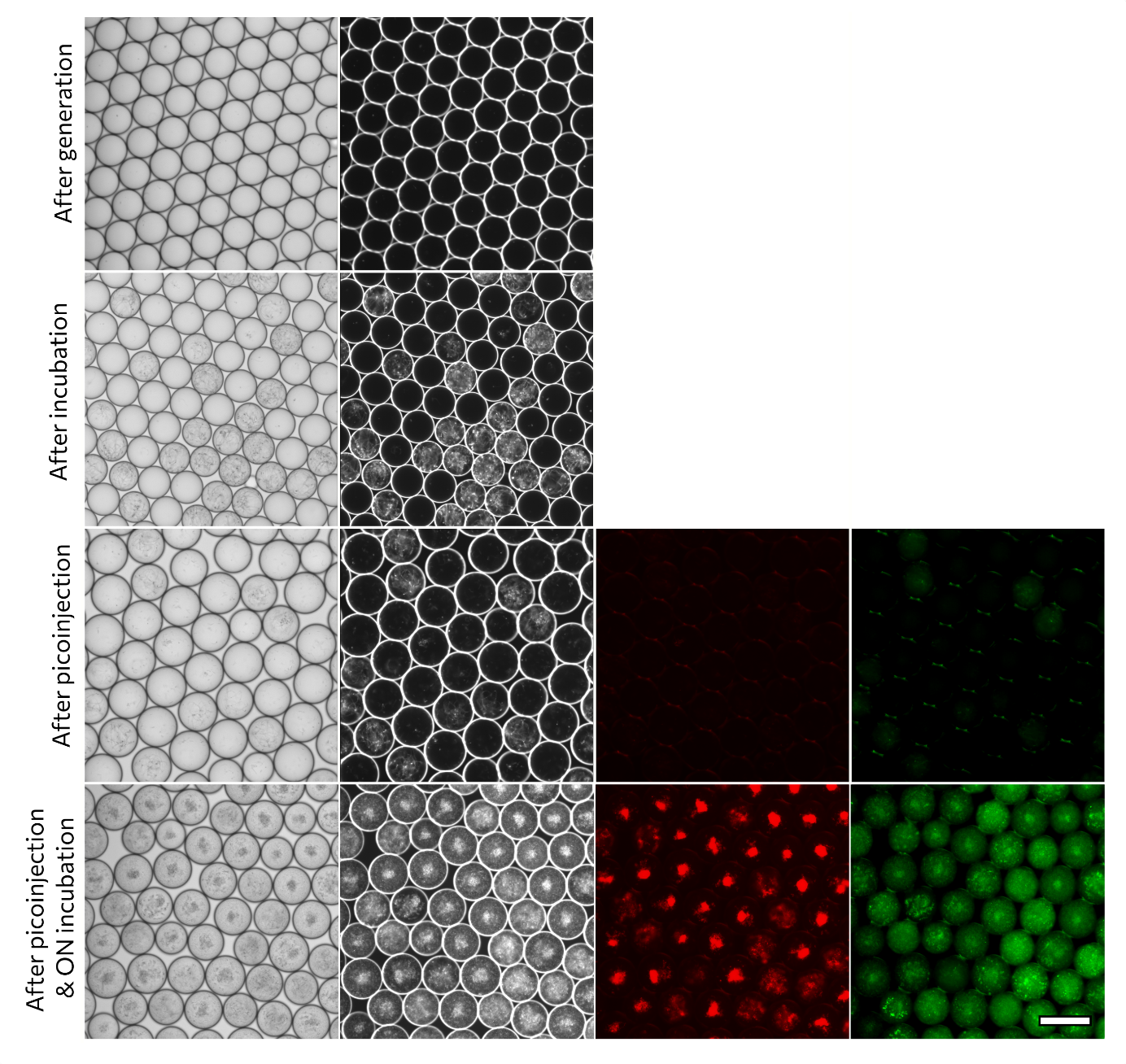


Fig. S8. Images of droplets during screening of model library. Droplets were generated at λ = 0.4. Dense mycelial growth is observed after incubation for 4 days. A mixture of reporter strains (at 20:80 ratio of *E. coli* EC081 and *B. subtilis* BS168) is picoinjected and further incubated. Increase in red (indicating growth of *B. subtilis*) and green (indicating growth of *E. coli*) fluorescence is observed in many droplets, while some droplets showed selective inhibition of either one or both reporter strains. Scale bar is 100 µm.


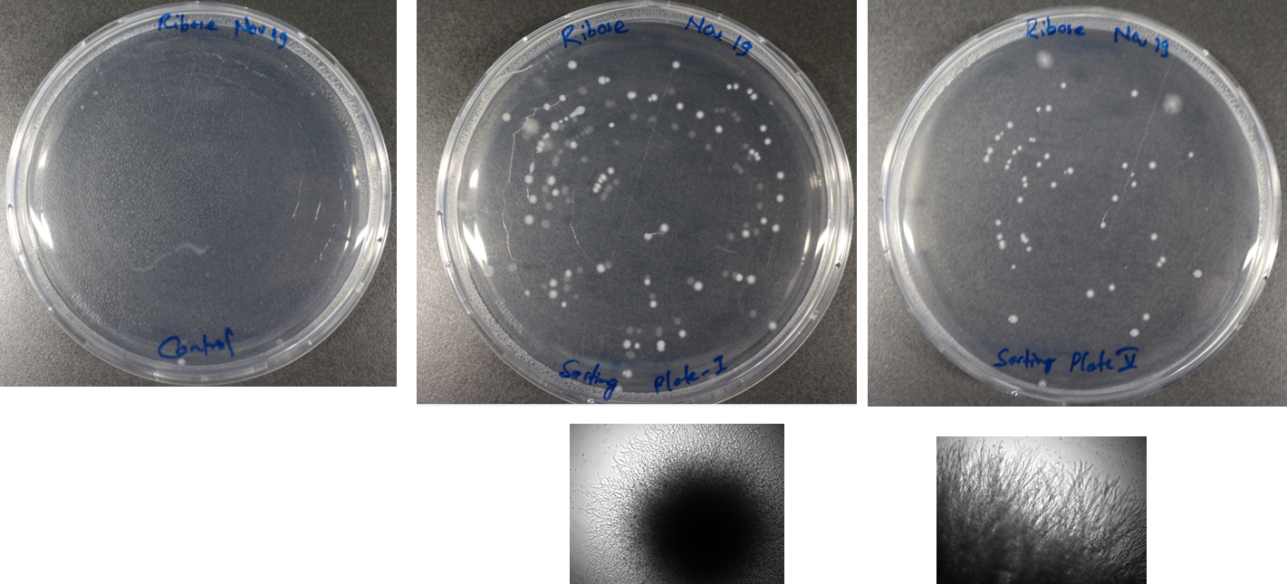


Fig. S9. Recovery of sorted droplets from model library on agar plates of MM+ribose (upper panel). Only representative agar plate images are shown. Control plate shows no colonies, while plates with dispensed droplets (center and right) resulted in growth of several colonies. Lower panel shows representative microscopic images of *Streptomyces* colonies showing hyphae and spores.

# Selection of agar media for growth of *Streptomyces*

Agar plates with different carbon sources (as listed in supplementary table S1) were prepared. 0.2 µl of *Streptomyces* (*S. noursei* SM2-2) droplets (grown for 3 days, picoinjected with both reporter strains, and incubated for 24 h) along with 50 µl of carrier oil were poured onto an agar plate of the medium as mentioned above in Table S1. The plate was tilted and rotated for spreading droplets. These plates were incubated for 3 days at 28 °C. The growth of microorganisms was determined by counting the number of colonies and checking colony morphology under a microscope and fluorescence under UV light (Fig. S10).


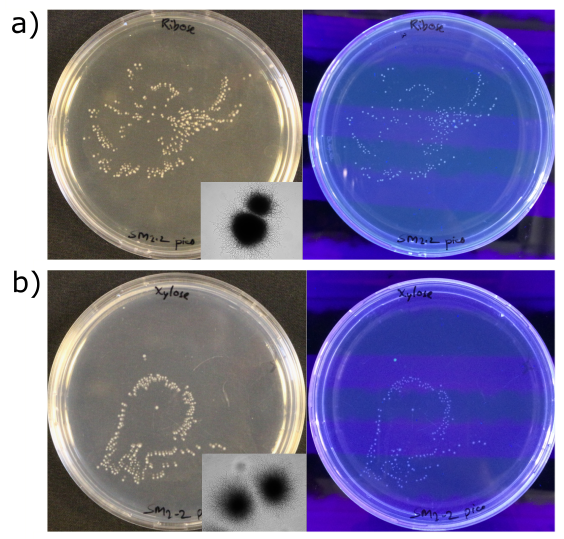


Fig. S10. Agar plates with colonies of *S. noursei* SM2-2 droplets in a) MM+Ribose medium and b) MM+Xylose medium. The right panel images are taken under UV light, to differentiate colonies of reporter strains from *Streptomyces*. The inset picture shows a microscopic image of colonies.

# Validation of sorted droplets from model library

*S. noursei* producing nourseothricin harbor the nourseothricin acetyltransferase gene *nat1* conferring self-resistance against the own antibiotic^6^. The gene sequence for *nat1* of *S. noursei* was accessed from the European Nucleotide Archive (Sequence: X73149.1). Primers specific for the *nat1* gene were designed by using Primer-BLAST (NCBI), yielding a product length of 198 bases. Sequences of forward and reverse primers are listed below.

Forward: CGAACAGTCGTCGAAATGGG

Reverse: GGTAAGCCGTGTCGTCAAGA

The *nat1* gene was amplified from colonies of different *Streptomyces* strains using the primers as mentioned above and a PCR Master Mix (Dreamtaq HS Green Master Mix, Thermo). The PCR was carried out as follows: pre-denaturation at 95 °C for 5 min, 35 cycles of denaturation at 95 °C for 30 s, annealing at 50 °C for 30 s, elongation at 72 °C for 30 s, and final elongation at 72 °C for 10 min. The length of the amplified gene was verified by comparing it with a DNA ladder (Fig. S11). The gene band of ~198 bases was observed only in *S. noursei*. Using the same primer pair, colony PCR was performed for isolates recovered from droplet screening of model library containing 5 different *Streptomycetes* (Fig. S12).


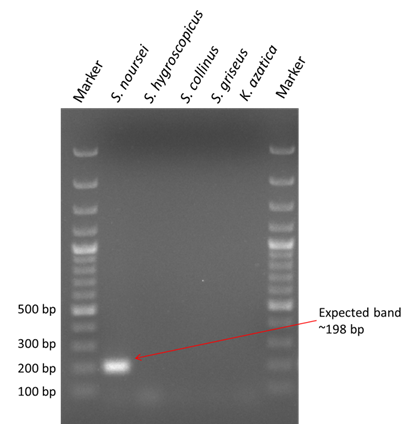


Fig. S11. Analysis of the amplified DNA fragments of the *nat1* gene. Only from *S. noursei* colonies, the expected band at ~198 bp was amplified.


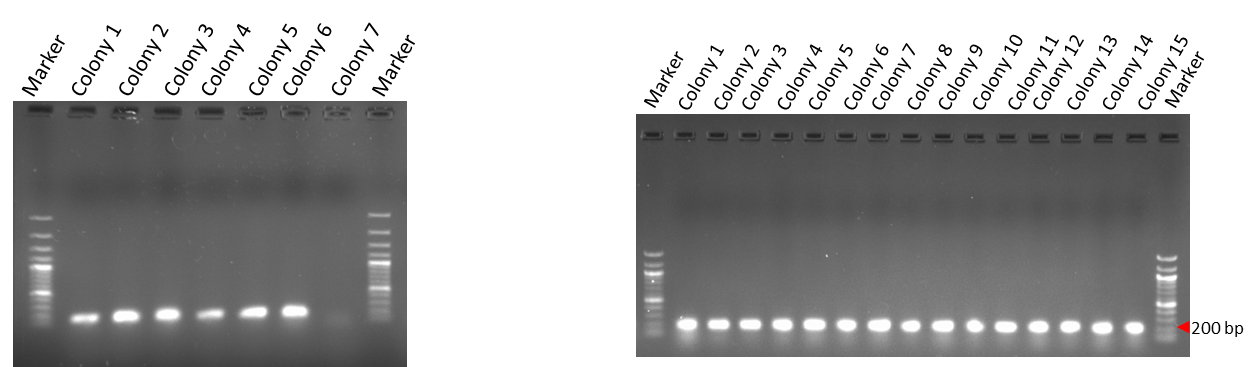


Fig. S12 Sample gel images of randomly selected colonies from model library screening, showing presence of expected band at ~198 bp. In some colonies, no band was observed, as these colonies might have grown from reporter strains.

# Droplet screening of environmental soil

Three environmental soil samples from different locations (natural reserve area soil – marked as SB, garden soil – marked as GS, and tar-contaminated soil – marked as TC) were used for microbial cells extraction. These extracted cells were encapsulated in droplets and incubated, resulting in growth of microorganisms with diverse morphology (Fig. S13). A mixture of reporter strains (20:80 ratio of EC081 and BS168) was picoinjected to droplets from SB samples and incubated. Similarly, for droplets of GS and TC samples, a mixture of reporter strains (20:80 ratio of EC081 and BS3610) were used. Microscopy images (Fig. S14) and fluorescence intensities (Fig. S15) were obtained and utilized for thresholding during sorting experiments. Sorted colonies were dispensed on NBA agar plates for colony recovery and isolation (Fig. S16).

**
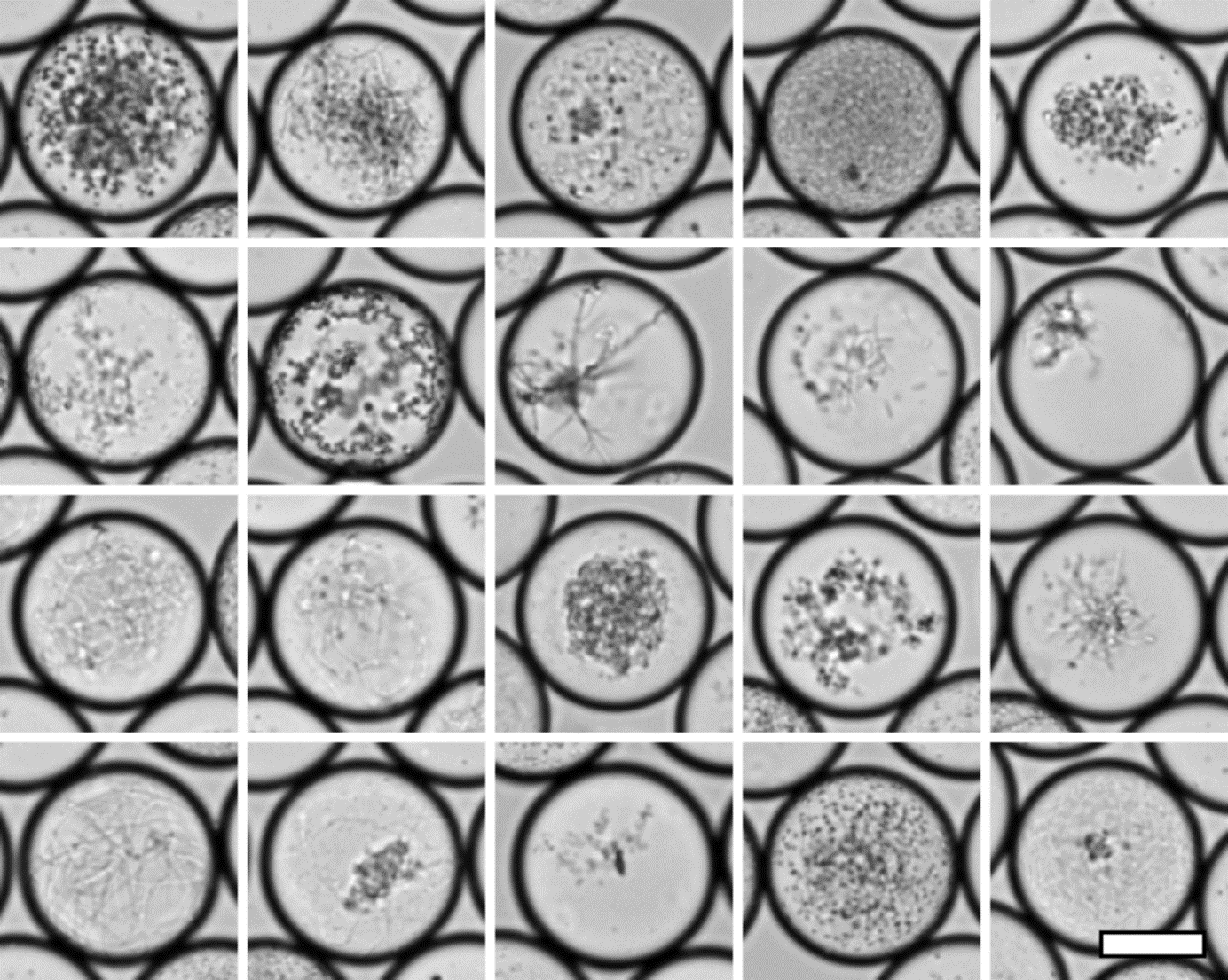
**

Fig. S13 Bright-field images of selected droplets showing growth of environmental microorganisms with different morphology. Scale bar is 50 µm.


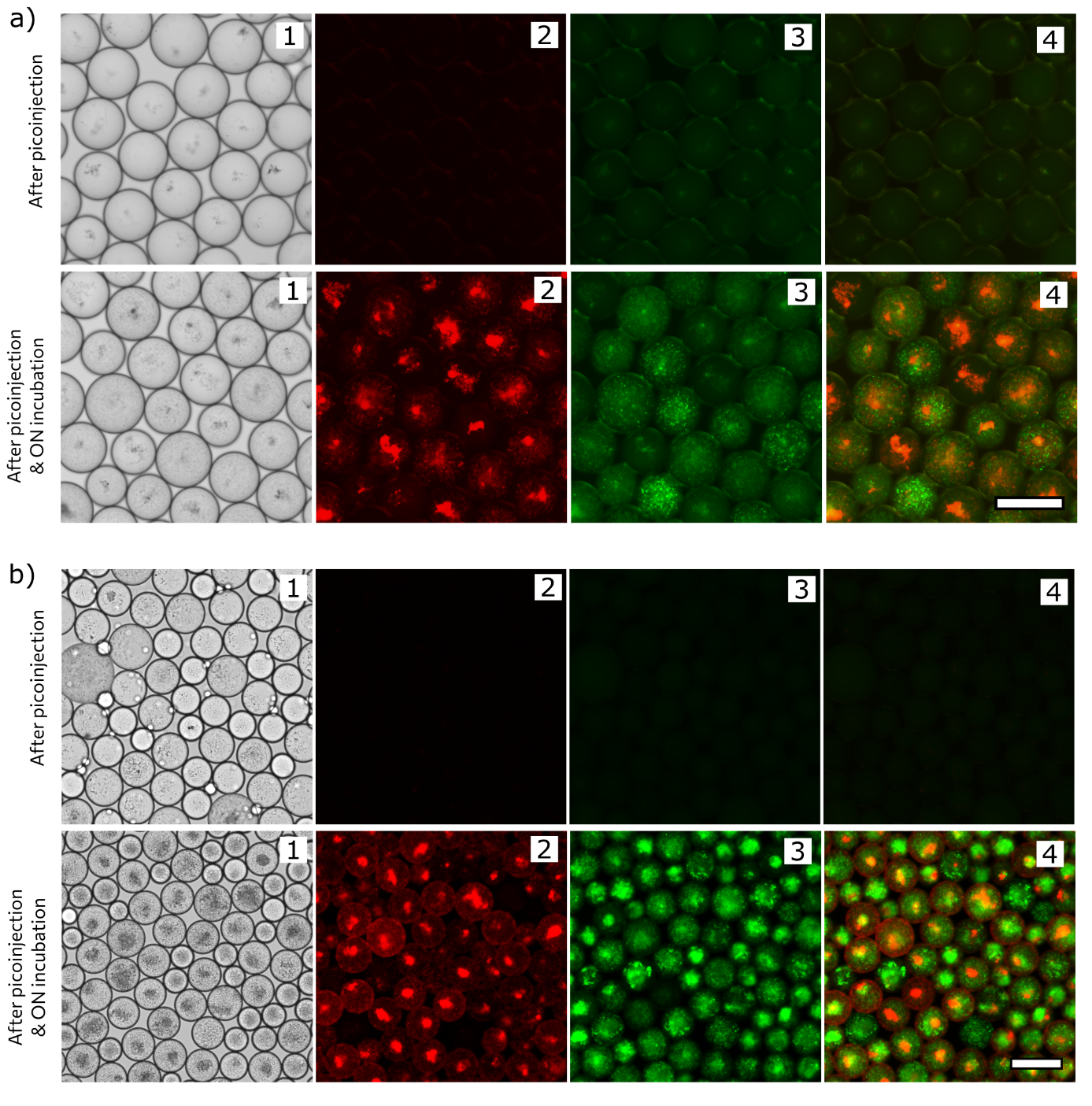


Fig. S14 Images of droplets for monitoring growth of reporter strains. After picoinjection of mixed *E. coli*/*B. subtilis* reporter strains and further overnight (ON) incubation, increase in red and green fluorescence intensities are observed. (a) For the natural reserve area soil (SB) sample and (b) garden soil (GS) sample. 1-Bright-field, 2-red (= *B. subtilis*), 3-green (= *E. coli*) and 4-overlay of red and green fluorescence channel. Scale bar is 100 µm.


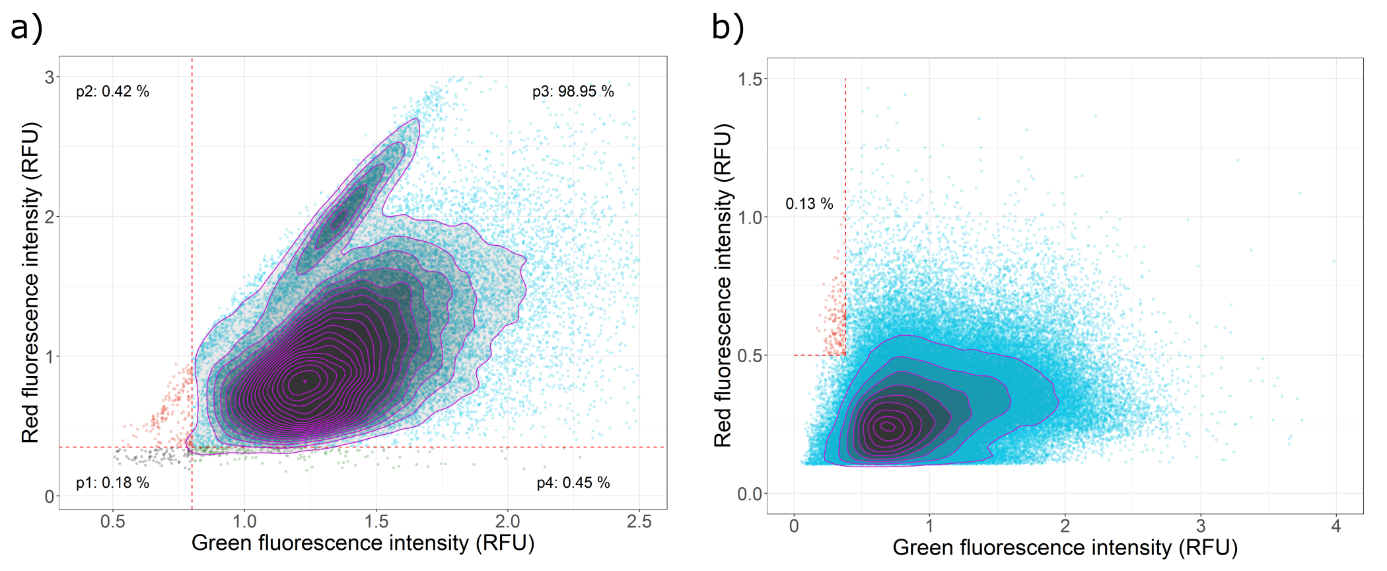


Fig. S15 Fluorescence signal measurement using our optofluidic platform during sorting of environment samples: (a) natural reserve area soil (SB) sample and (b) garden soil (GS) sample. Red fluorescence intensity represents growth of the *B. subtilis* mKate reporter strain; green fluorescence intensity represents growth of the *E. coli* GFP reporter strain within the environmental droplets. Each dot represents a droplet. Red dotted lines represent the thresholds for red and green fluorescent channels for sorting. Relative frequency of selected droplet is specified in percentage. For the SB sample (a), sorting targeted broadband antibiotic activity (low red and low green signal) and for the GS sample (b), sorting targeted anti-Gram-negative bioactivity (low green signal for *E. coli*).


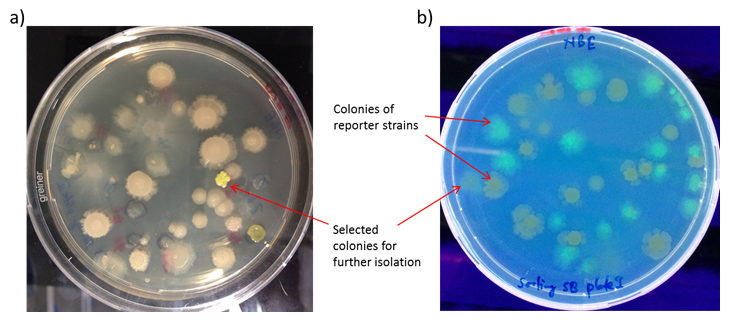


Fig. S16. Agar plates with colonies grown from screening of environmental soil samples. a) Plate image showing selected colonies for further isolation. b) Plate image under UV light showing colonies of reporter strains isolated along with other environmental cells. Selected colonies were re-streaked to obtain a pure culture colony.

# Validation of isolated colonies from droplet screening of environmental soil

Recovered isolates from droplet screening of environmental soil samples were cultivated in SM media for 7 days. A small volume of culture suspension was aliquoted and marked as ‘cell suspension’. From the remaining culture suspension, pellets and supernatants were collected by centrifugation at 15000 xg for 15 min. All these three samples (cell suspension, pellet and supernatant) were stored at -20 °C until further processing. Cell suspension and supernatants were used for inhibition assay of reporter strains in microtiter well plates using Biolector system (Fig. S17). Additionally, pellets and supernatants were used for Methanol and Ethyl-acetate extraction respectively. These extracts along with supernatants were used for agar-diffusion bioactivity assay (Table S3).


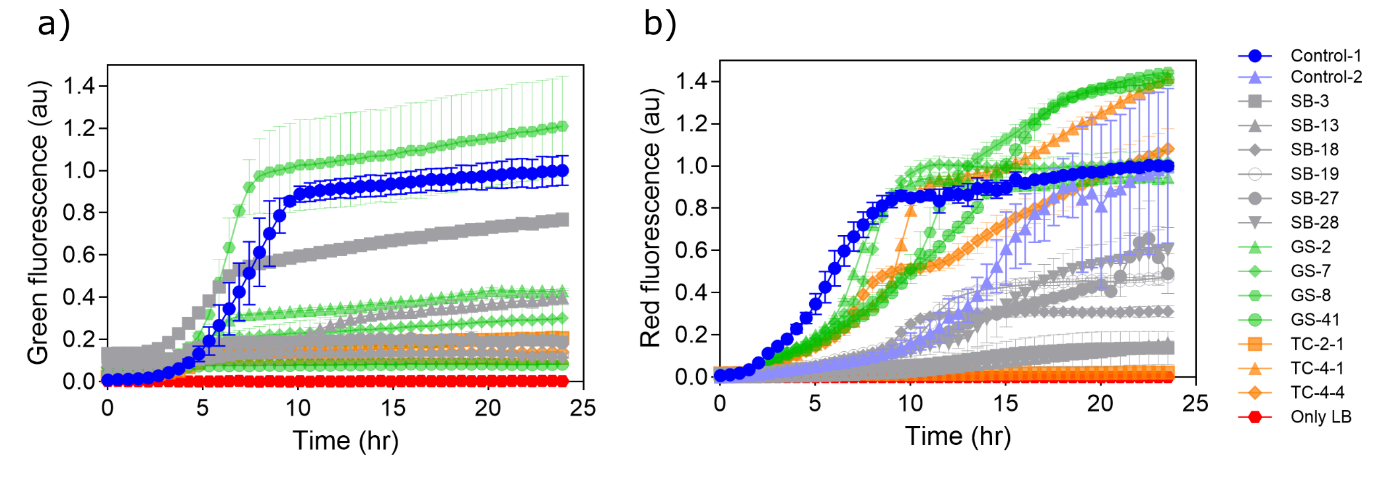


Fig. S17. Inhibition of reporter strains by supernatants of recovered isolates in microtiter well plates. Growth of reporter strains was quantified by monitoring fluorescence in green (a) for *E. coli* EC081 and in red (b) for *B. subtilis*. *B. subtilis* strain BS168 was used to test isolates from the SB sample (colored grey), while strain BS3610 was used for GS (colored light green) and TC (colored light orange) samples. Wells containing growth media with reporter strain are used as controls (Control-1 for BS3610 and Control-2 for BS168).

Table S3-a/b/c. Results of standard bioactivity assays of environmental isolates. Data in table represent the size of halo regions in an agar-diffusion assay with a panel of selected microorganisms (top row). Strain ID for each microorganism represents JMRC identification numbers. The assay was performed with a) supernatant, b) methanol extract or c) ethyl acetate extract of isolates from soil samples (SB: from natural reserve area, TC: tar-contaminated, GS: garden soil). All measurements are in millimeters. Inhibition controls (Ref media: reference media, Cip: ciprofloxacin 5 µg/ml, Ampho B: amphothericin B 10 µg/ml) are listed at the bottom of each table. Abbreviations - p: partially clear (< ~50%) halo region, P: partially clear (between 50% to 100%) halo region, p-P: halo region between p and P, EK: clear halo with few colonies, A: ambigious (maybe small halo but not clear).

| a) Super-natant | | *Bacillus spiziziensis* | *Staphylococcus aureus* | *Escherichia coli* | *Pseudomonas aeruginosa* | *Mycobacterium vaccae* | *Sporobolomyces salmonicolor* | *Candida albicans* | *Penicillium notatum* | *E. coli* | *B. subtilis* | *B. subtilis* |
| --- | --- | --- | --- | --- | --- | --- | --- | --- | --- | --- | --- | --- |
|  |  | ATCC6633 | SG511 | SG458 | K799/61 | 10670 | 549 | CA | JP36 | EC081 | BS168 | BS3610 |
| 1 | SB-3 |  |  |  |  | 0/A |  |  |  |  |  |  |
| 2 | SB-13 |  |  |  |  |  | 0/A |  |  |  |  |  |
| 3 | SB-18 |  |  |  |  |  |  |  |  |  |  |  |
| 4 | SB-19 |  |  |  |  |  |  |  |  |  |  |  |
| 5 | SB-27 |  |  |  |  |  |  |  |  |  |  |  |
| 6 | SB-28 |  |  |  |  |  |  |  |  |  |  |  |
| 7 | TC-2-1 | 11/18p | 10/18p-P |  |  | 10/17p |  |  | 11P | 19P | 20P | 10/17p |
| 8 | TC-4-1 |  |  |  |  |  |  |  |  |  |  |  |
| 9 | TC-4-4 |  |  |  |  |  |  |  |  |  |  |  |
| 10 | GS-2 |  |  |  |  | 12p | 0/A |  | 13P |  |  | 0/A |
| 11 | GS-7 |  |  |  |  | 11 |  |  |  |  |  |  |
| 12 | GS-8 |  |  |  |  |  |  |  |  |  |  |  |
| 13 | GS-41 |  |  |  |  |  |  |  |  |  |  |  |
|  | Ref media |  |  |  |  |  |  |  |  |  |  |  |
|  | Cip | 28 | 20 | 24/31p | 28/34p | 22p |  |  |  | 30 | 28/30EK | 29/31EK |
|  | Ampho B |  |  |  |  |  | 17p | 21 | 18p |  |  |  |
|  |  |  |  |  |  |  |  |  |  |  |  |  |
| b) Methanol extract | | *Bacillus spiziziensis* | *Staphylococcus aureus* | *Escherichia coli* | *Pseudomonas aeruginosa* | *Mycobacterium vaccae* | *Sporobolomyces salmonicolor* | *Candida albicans* | *Penicillium notatum* | *E. coli* | *B. subtilis* | *B. subtilis* |
|  |  | ATCC6633 | SG511 | SG458 | K799/61 | 10670 | 549 | CA | JP36 | EC081 | BS168 | BS3610 |
| 1 | SB-3 | 13/17p-P | 11/15P |  |  | 18p |  |  |  | 10 | 14 | 13/15EK |
| 2 | SB-13 | 10 |  |  |  |  |  |  |  |  |  |  |
| 3 | SB-18 | 10 |  |  |  |  |  |  |  |  |  |  |
| 4 | SB-19 | 10 |  |  |  |  |  |  |  |  |  |  |
| 5 | SB-27 | 10 |  |  |  |  |  |  |  |  |  |  |
| 6 | SB-28 | 10 |  |  |  |  |  |  |  |  |  |  |
| 7 | TC-2-1 | 13/19P/35p-P | 13/35p |  |  | 24p/35P |  |  |  | 10 | 18/20EK | 15/17EK |
| 8 | TC-4-1 | 10 |  |  |  |  |  |  |  |  |  |  |
| 9 | TC-4-4 | 10 |  |  |  |  |  |  |  |  |  |  |
| 10 | GS-2 | 0/A |  |  |  | 12p-P |  |  |  |  |  |  |
| 11 | GS-7 | 10 |  |  |  | 13p |  |  |  |  |  |  |
| 12 | GS-8 | 10 |  |  |  | 14p |  |  |  |  |  |  |
| 13 | GS-41 | 10 |  |  |  |  |  |  |  |  |  |  |
|  | Ref MeOH |  |  |  | 11P |  | 10 |  | 10 |  |  |  |
|  | Cip | 26 | 19 | 24/31p | 28/35p | 20p |  |  |  | 30 | 29/32EK | 27/30EK |
|  | Ampho B |  |  |  |  |  | 17p | 21 | 18p |  |  |  |
|  |  |  |  |  |  |  |  |  |  |  |  |  |
| c) Ethyl Acetate extract | | *Bacillus spiziziensis* | *Staphylococcus aureus* | *Escherichia coli* | *Pseudomonas aeruginosa* | *Mycobacterium vaccae* | *Sporobolo-myces salmonicolor* | *Candida albicans* | *Penicillium notatum* | *E. coli* | *B. subtilis* | *B. subtilis* |
|  |  | ATCC6633 | SG511 | SG458 | K799/61 | 10670 | 549 | CA | JP36 | EC081 | BS168 | BS3610 |
| 1 | SB-3 | 13/17p/21P/27p | 13/30P |  |  | 18p | 12P |  | 0/A | 10 | 15 | 15/17p |
| 2 | SB-13 | 10 |  |  |  |  |  |  |  |  |  |  |
| 3 | SB-18 |  |  |  |  |  |  |  |  |  |  |  |
| 4 | SB-19 | 10 |  |  |  |  |  |  |  |  |  |  |
| 5 | SB-27 | 11EK |  |  |  |  |  |  |  |  |  |  |
| 6 | SB-28 | 11EK |  |  |  |  |  |  |  |  |  |  |
| 7 | TC-2-1 | 14/36p | 13/40p |  |  | 18/37p |  |  | 0/A | 10/25P | 15/25p | 15/22p |
| 8 | TC-4-1 | 11EK |  |  |  |  |  |  |  |  |  | 10 |
| 9 | TC-4-4 | 11EK |  |  |  |  |  |  |  |  |  |  |
| 10 | GS-2 | 11EK |  |  |  | 11p |  |  |  | 0/A |  |  |
| 11 | GS-7 | 11EK |  |  |  |  |  |  |  |  |  |  |
| 12 | GS-8 | 10EK |  |  |  |  |  |  |  |  |  |  |
| 13 | GS-41 |  |  |  |  |  |  |  |  |  |  |  |
|  | Ref EA |  |  |  | 11P |  |  |  |  |  |  |  |
|  | Cip | 28 | 19 | 23/31p | 27/34p | 20p |  |  |  | 30 | 28/31EK | 29/31EK |
|  | Ampho B |  |  |  |  |  | 17p | 21 | 18p |  |  |  |

# HPLC-HRMS analysis of extracts

One selected isolate from environmental screening (SB-3) was cultivated in 10 mL of SM media in 50 mL shake flask for 7 days. Supernatant was collected by centrifugation at 10000 xg for 15 min and used for Ethyl-acetate extraction. LC/MS analysis of the extract was performed using an Exactive Orbitrap high performance benchtop LC-MS (Thermo Fisher Scientific) with an electron spray ion source and an Accela HPLC system with a photodiode array detector: C18 column (Betasil C18 5 µm, 150 × 2.1 mm, Thermo Fisher Scientific), solvents: acetonitrile and water (both supplemented with 0.1 % formic acid), at flow rate: 0.2 mL min^-1^; program: hold 1 min at 5 % acetonitrile, 1–16 min 5–98 % acetonitrile, hold 3 min 98 % acetonitrile, afterwards re-equilibration to starting conditions. A compound with *m*/*z* 515.3333 ([M+H]^+^, calcd. for C_26_H_47_N_2_O_8_ *m/z* 515.3327) along with several congeners were detected that correspond to the molecular composition of serratamolides (Fig. S18-S20).

Detected derivatives:

*m/z* 515.3333 [M+H]^+^, calcd. for C_26_H_47_N_2_O_8_ *m/z* 515.3327

*m/z* 533.3442 [M+H]^+^, calcd. for C_26_H_49_N_2_O_9_ *m/z* 533.3433

*m/z* 529.3496 [M+H]^+^, calcd. for C_27_H_49_N_2_O_8_ *m/z* 529.3483

*m/z* 541.3492 [M+H]^+^, calcd. for C_28_H_49_N_2_O_8_ *m/z* 541.3483

*m/z* 543.3642 [M+H]^+^, calcd. for C_28_H_51_N_2_O_8_ *m/z* 543.3640

Fig. S18. Structure of serratamolide A and list of detected derivatives


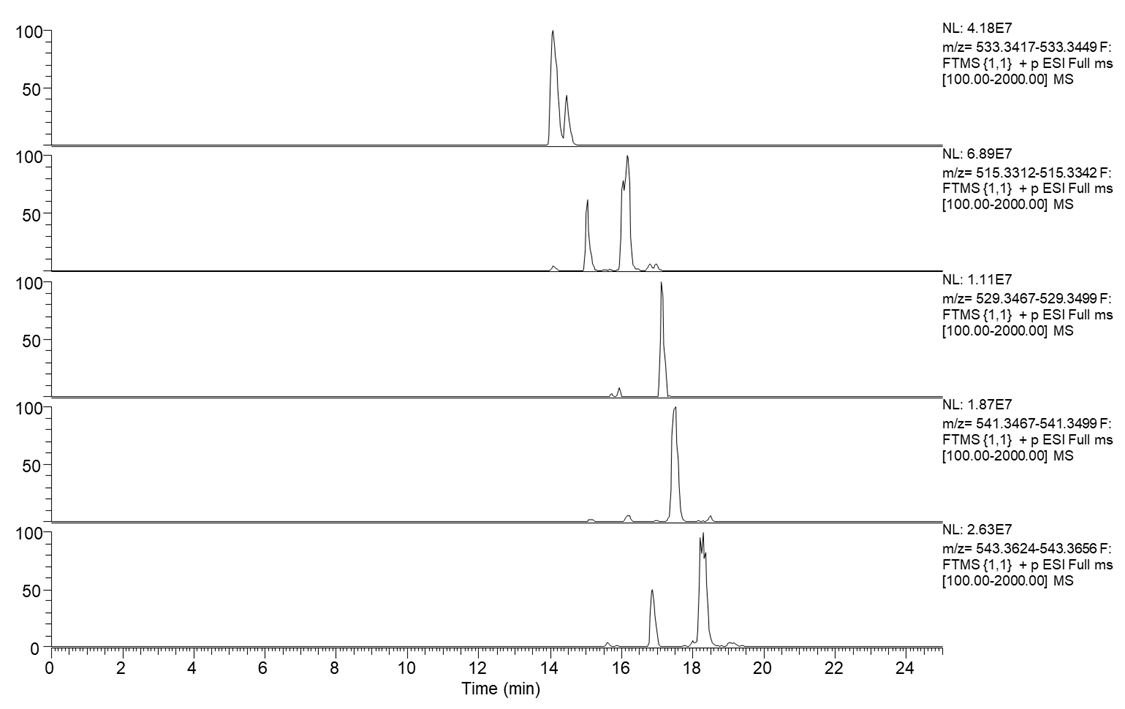


Fig. S19. HPLC-HRESI-MS profiles of SB-3 extract. The extracted ion chromatograms are shown (positive ionization mode).

a)
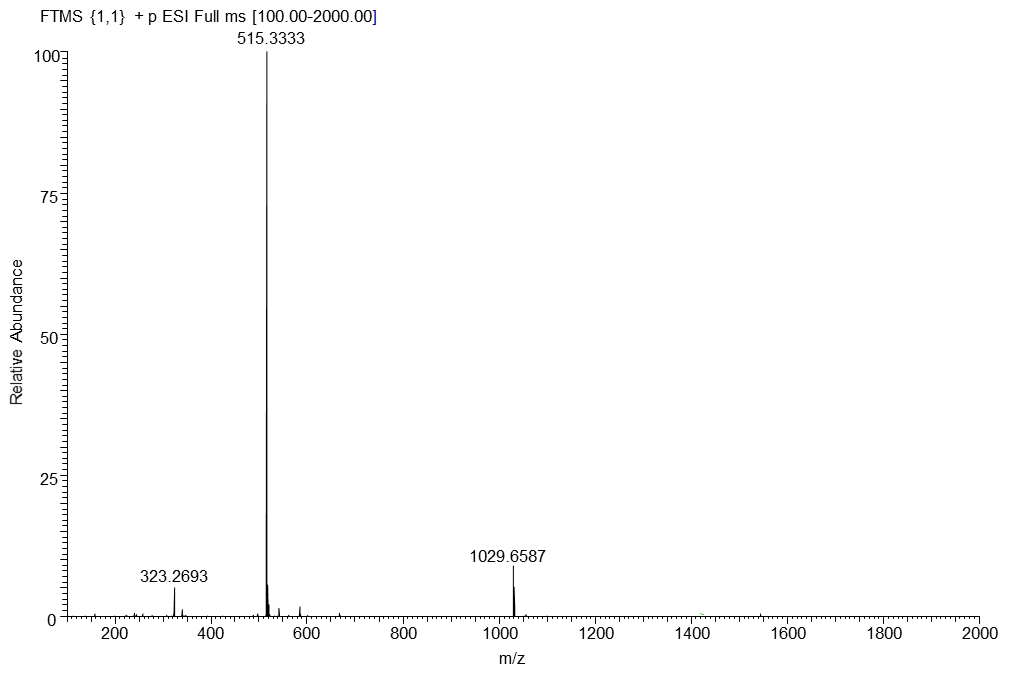


b)
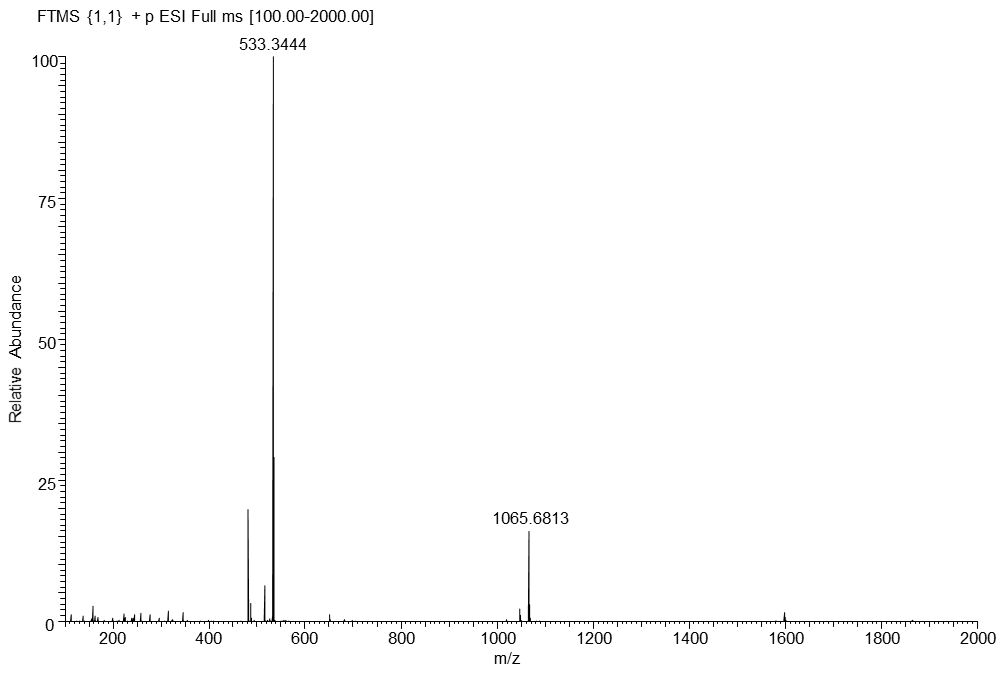


c)
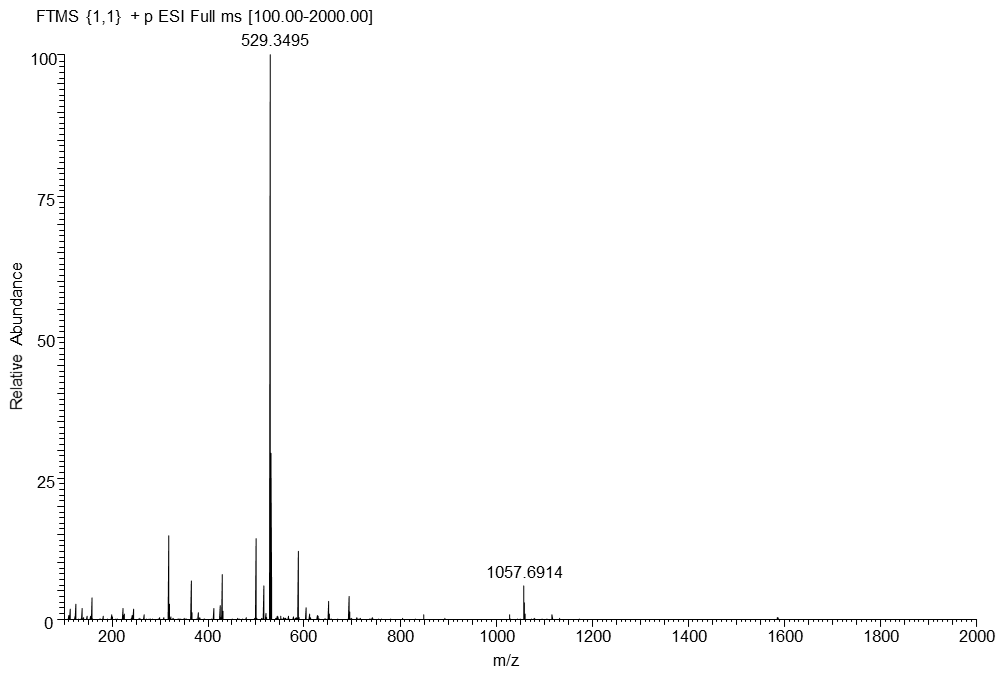


d)
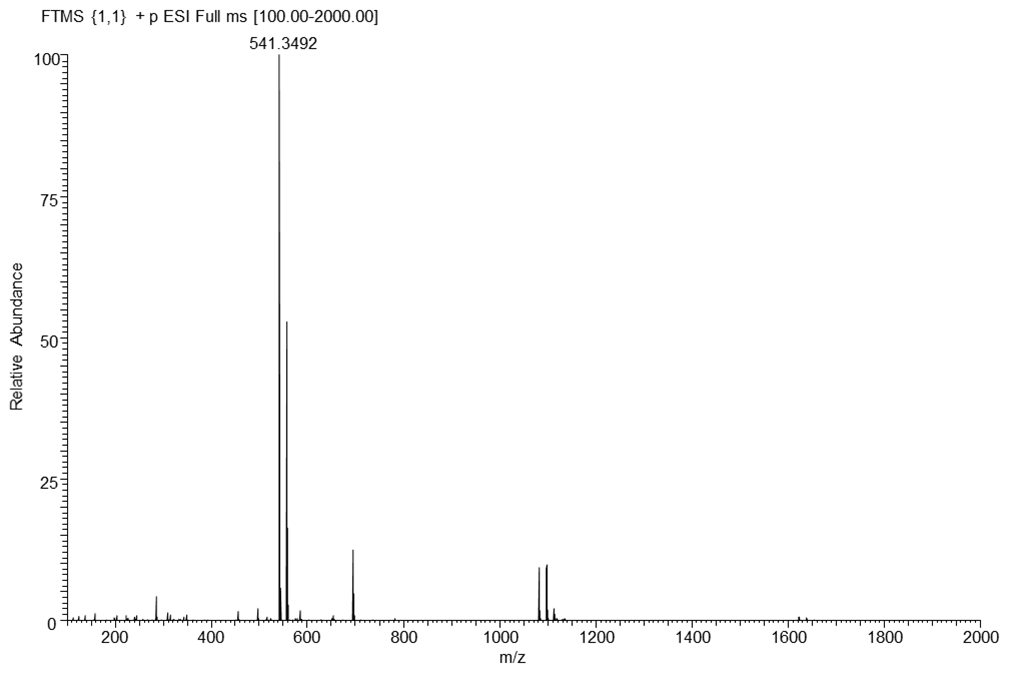


e)
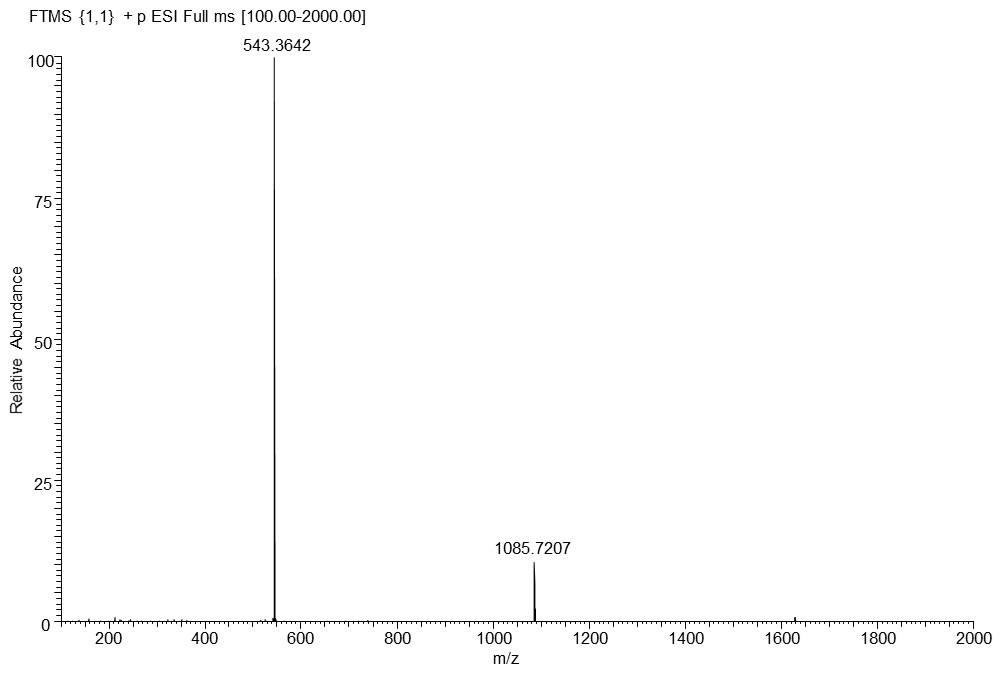


Fig. S20. HRESI(+)-MS spectra of five detected metabolites. The m/z value of the major ion in the main peak is shown.

# Supplementary technical methods

## Picoinjection process

For optimizing the picoinjection process, a new microfluidic structure was designed and fabricated. The width of the microfluidic channel was gradually decreased near the picoinjection nozzle to squeeze the droplets (Fig. S21 a and b). Squeezing of droplets provided extended residence time of droplets near the picoinjection nozzle, which ultimately resulted in improved picoinjection efficiency even for droplets of various sizes (Fig. S21c). The efficiency of simultaneous picoinjection of both reporter strains was quantified by measuring the variability of green and red fluorescence intensities of droplets before and after incubation. A mixture of reporter strains (20:80 ratio of *E. coli* EC081 and *B. subtilis* BS168) was picoinjected into medium droplets (frequency 300 droplets/second). Picoinjected droplets were incubated for 24 h, and fluorescence intensities were measured. More than 99 % of droplets showed high fluorescence in both green and red channels demonstrating effective simultaneous picoinjection of both reporter strains (Fig. S21 d and e).


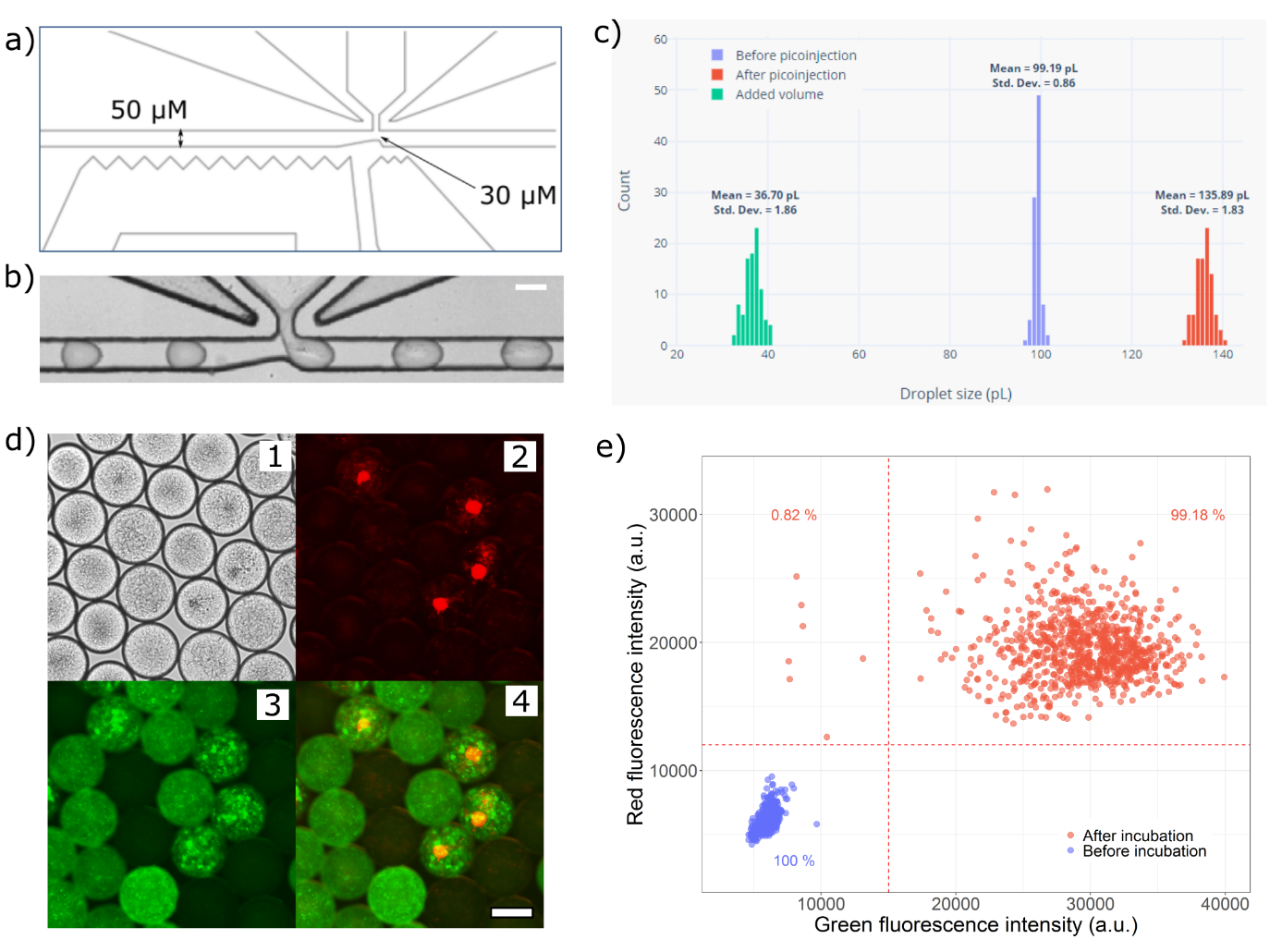


Fig. S21. Evaluation of picoinjection efficiency. a) Design parameters near the picoinjection nozzle showing the squeezed fluidic channel and electrodes. b) Microscopic image of the picoinjection nozzle during picoinjection. c) Analysis of picoinjection volume by image analysis. Sizes of droplets were determined before and after picoinjection and used for calculating the picoinjected volume. d) Droplets containing a mixture of the reporter strains were picoinjected into medium droplets and incubated for 24 h. Images of droplets showing growth of both reporter strains. 1- bright-field, 2-red, 3- green, 4-overlay of green and red fluorescence images. e) Comparison of fluorescence intensities before and after cultivation. Fluorescence intensity values were derived from image analysis. Red dotted lines represent thresholds for red and green fluorescence for differentiating droplet populations. Each data point represents one droplet. Scale bar is 50 µm.

Additionally, the efficiency of picoinjection was quantified by image analysis of droplets before and after picoinjection. Images of picoinjection were taken by using a fast camera at 800 fps, such that droplets could be tracked during picoinjection (Video S1). The size of the droplet was measured by detecting the droplet borders. For each droplet, the picoinjected fraction was determined by subtracting the volume of the droplet before picoinjection from the volume of the droplet after picoinjection. The amount of picoinjected volume for different droplet samples (droplets with and without cells) was determined. Droplets were picoinjected efficiently with minimal variation (Fig. S21c).


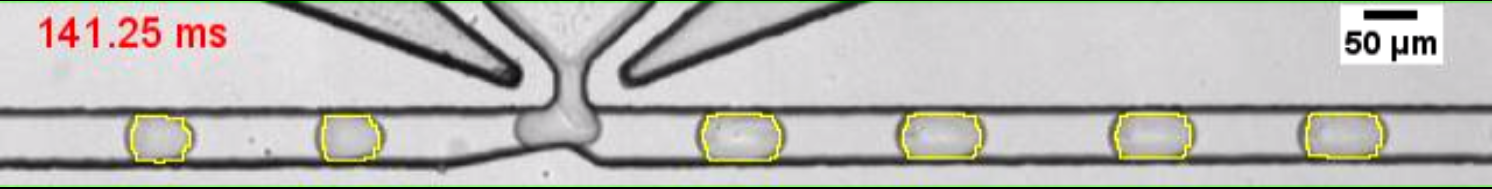


Video S1. Tracking of droplets during picoinjection for image analysis.

## Sorting setup and electronics

The sorting process was optimized by improving the signal analysis algorithm and implementing a droplet collection structure on the chip for analyzing sorted droplets. Droplets with inhibited growth of reporter cells have lower fluorescence intensities. This makes it difficult to detect and analyze droplets with a conventional setup, which generally considers higher fluorescent signals exceeding the threshold values (in applications like finding improved strain variants in directed evolution). We implemented a multi-parametric approach in our optofluidic platform along with the Arduino-based sorting algorithm (Fig. S22). Either scattered signals or blue fluorescence signals (background fluorescence due to media components) were used as droplet marker signal. Once a droplet was detected with the droplet marker signal, fluorescence signals from reporter strains were analyzed. The Arduino was programmed to analyze multiple signals and generate sorting trigger signals allowing active sorting of droplets (Video S2). The Arduino code can be optimized for sorting of droplets depending on assay requirements with either both reporter strains inhibited (see code below) or only one inhibited. Additionally, to collect droplet populations sorted according to different criteria, a multi-level oval-shaped trap structure was integrated into the sorting structure^7^. This allowed not only real-time visualization of sorted droplets but also imaging and efficient recovery of sorted droplets in combination with the capillary-based positioning approach.


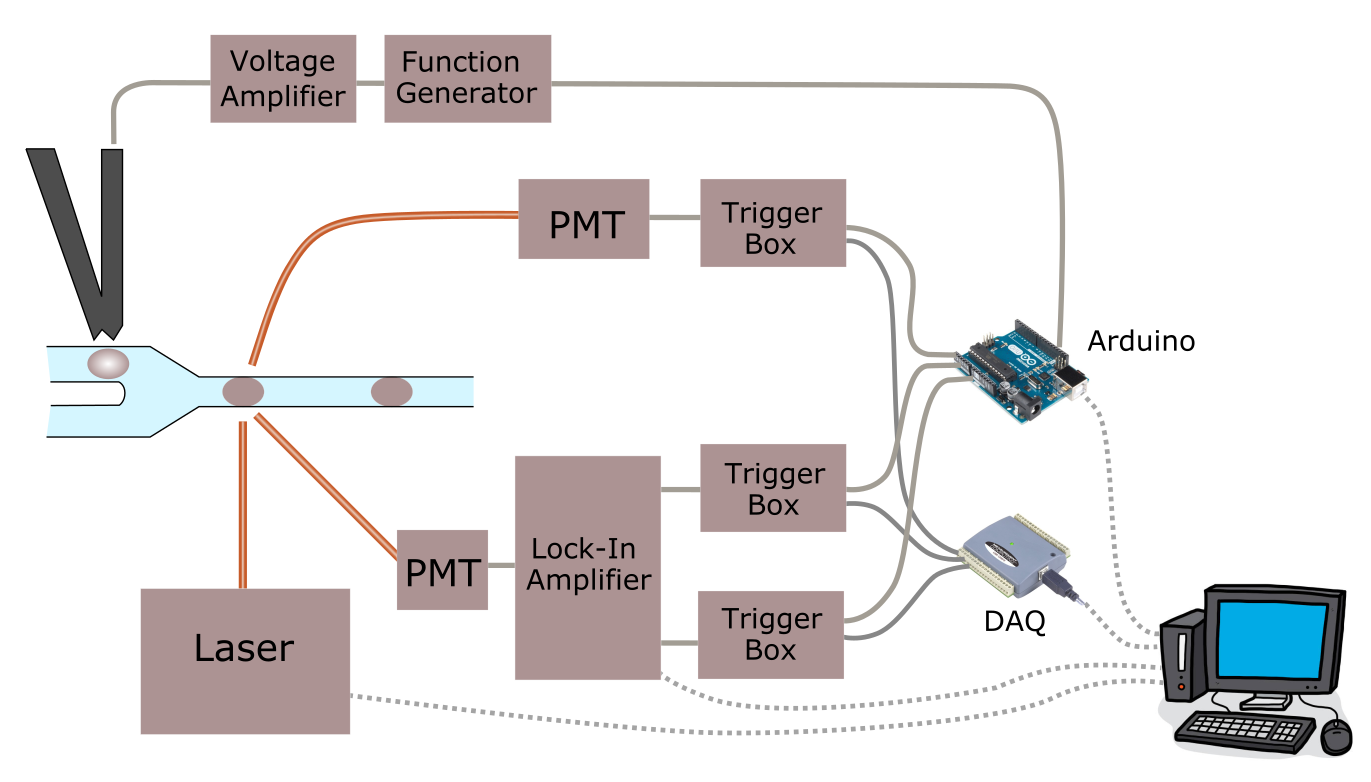


Fig. S22. Schematic showing the electronic setup for active sorting of droplets having low fluorescence intensities. An Arduino-based program was utilized to analyze trigger signals from different fluorescent channels and provide the trigger for sorting.


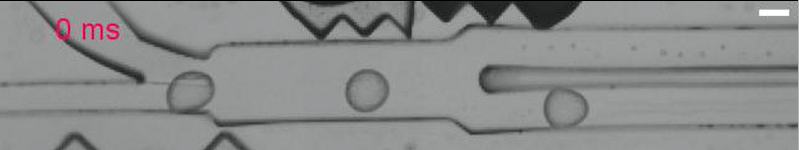


Video S2. Sorting of droplets based on optofluidic detection setup. Scale bar is 50 µm.

Arduino algorithm for actively sorting droplets with low fluorescence signals

//Scatter signal is a droplet identifier, red n green signal identifies (growth or) no growth

const int redChannel = 9;

const int greenChannel = 10;

const int scatterChannel = 8;

const int OutputPin = 5;

bool myGreen;

bool myRed;

bool myScatter;

void setup() {

pinMode(redChannel, INPUT);

pinMode(greenChannel, INPUT);

pinMode(scatterChannel, INPUT);

pinMode(OutputPin, OUTPUT);

}

void loop() {

if (digitalRead(scatterChannel) && myScatter == false) { // Detect start of the scatter pulse.

myScatter = true;

myGreen = false;

myRed = false;

}

if (digitalRead(redChannel) && myRed == false) { // Start of the red pulse. May or may not happen.

myRed = true;

}

if (digitalRead(greenChannel) && myGreen == false) { // Start of the green pulse. May or may not happen.

myGreen = true;

}

if (digitalRead(scatterChannel) == LOW && myScatter == true) { // End of the scatter pulse.

myScatter = false;

if (myRed == false && myGreen == false){ // there was no red and no green

delayMicroseconds(8000);

digitalWrite(OutputPin, HIGH);

delayMicroseconds(3000);

digitalWrite(OutputPin, LOW);

}

}

}

## Recovery of droplets

Droplets collected in the collection structure were deposited onto an agar medium in a Petri dish by using a capillary-based droplet positioning approach as explained previously^7^. Droplets were taken out of the chip through a capillary tube which was connected to the secondary exit channel (Fig. S23a). Positive pressure was applied to the exit channel of the collection chamber in a controlled way such that a single droplet is released at a time from a secondary outlet (Video S3). The capillary tube was fixed in an XYZ-positioning setup. A spiral movement was programmed to move the capillary in the XY plane with a fixed Z-axis. As the capillary moved on the surface of the agar plate, flowing droplets got positioned in spiral orientation. Agar plates were incubated, and the growth of microorganisms was determined by counting the number of colonies.

To verify the proper isolation of droplets, we performed a model experiment with droplets of *E. coli* ECJW992 producing the mCherry protein. Droplets from the collection structure were deposited on LB agar plates using the capillary-based method. After incubation, we observed 64 colonies out of 67 deposited droplets (Fig. S23b and c). All colonies had high red fluorescence.


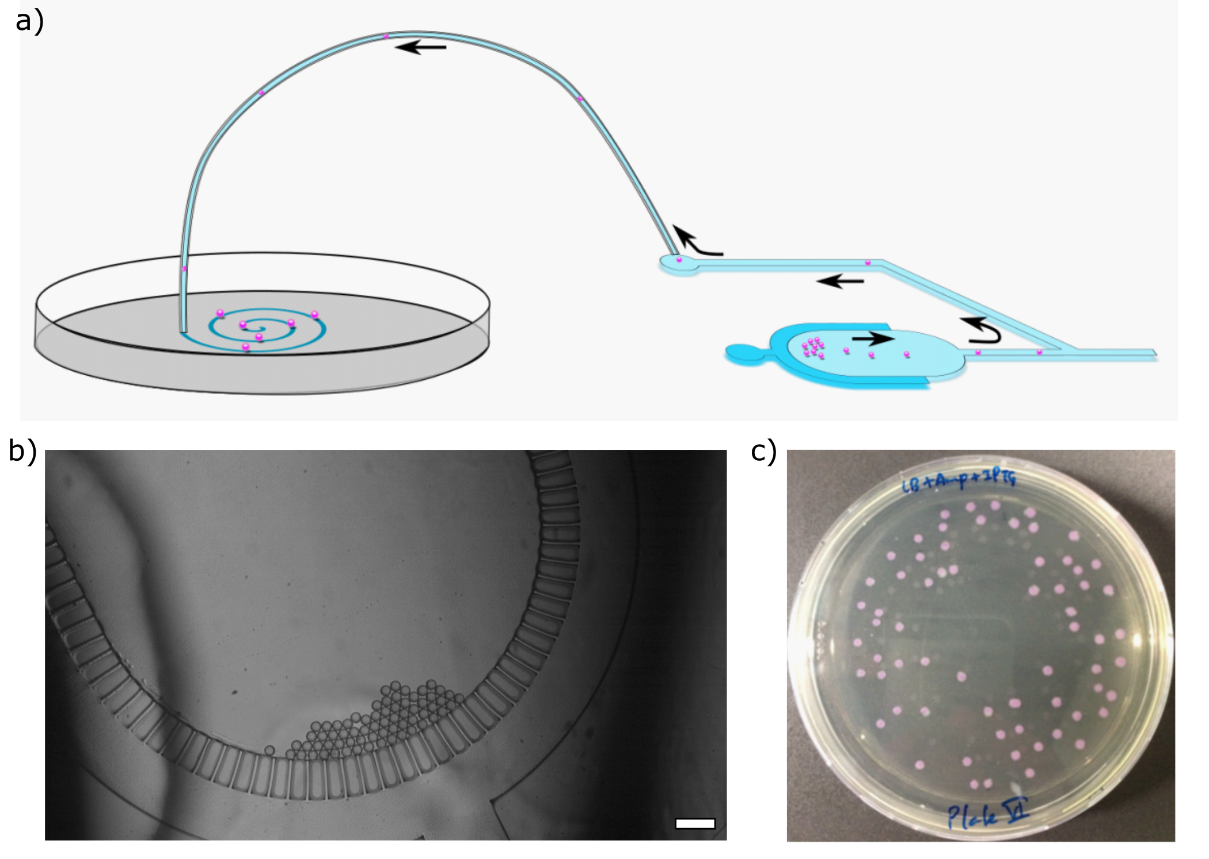


Fig. S23. Recovery and isolation of single droplets. a) Schematic showing the flow of droplets from the collection structure to an agar plate. Positive pressure is applied through the exit of collection chamber in a controlled manner to transfer a single droplet at a time into the capillary. The capillary tubing connected to the chip outlet is moved in spiral orientation using a positioning system. Droplets are deposited on the agar plate with spacing depending on the flow rate of the carrier oil. b) Image of sorted droplets in the collection chamber. c) Agar plate with colonies of test strain *E. coli* mCherry ECJW992^8^ grown from deposited droplets from image b. Each colony on the plate arises from a single droplet deposited using the described setup. The numbers of sorted droplets and grown colonies on agar plates were determined by image analysis and further confirmed by manual counting. Scale bar is 200 µm.


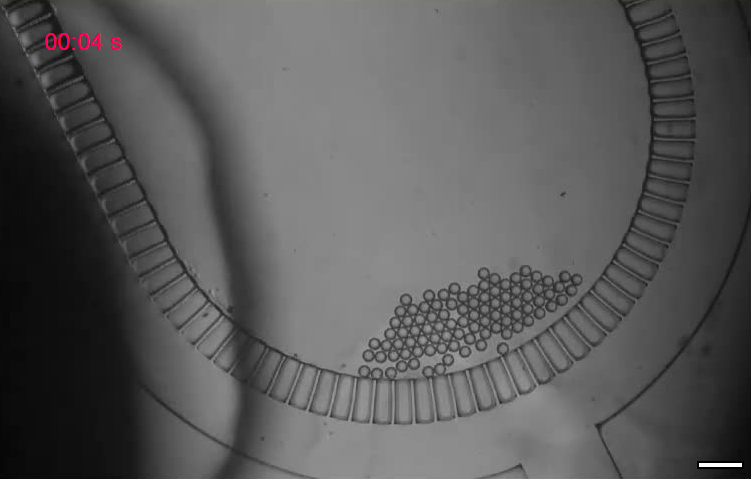


Video S3. Real-time video showing release of single droplet from collection chamber and dispensing on petri dishes. Scale bar is 200 µm.

## Microfluidic chip designs

All microfluidic chips were designed in AutoCAD software. Overview images with channel dimensions near droplet generation nozzle, picoinjection nozzle and sorting region are shown below. Additionally, respective AutoCAD files are also included as supplementary information.


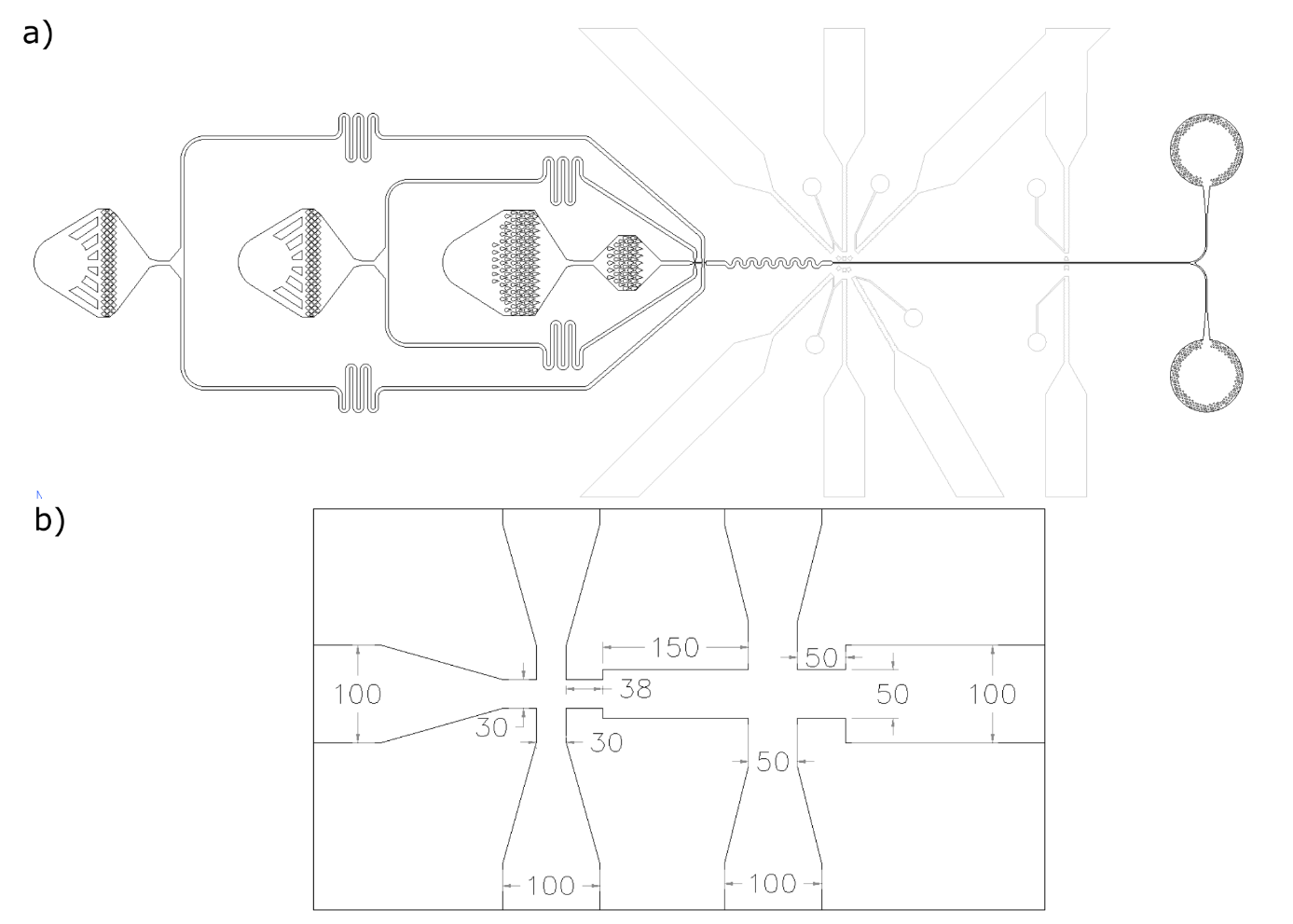


Fig. S24. Design of droplet generation chip. a) Overview image showing fluidic channels (in black) and optical fiber channels in gray (not used in this study). b) Zoom-in image showing channel dimensions at droplet generation nozzle. All annotations are in micrometer unit.


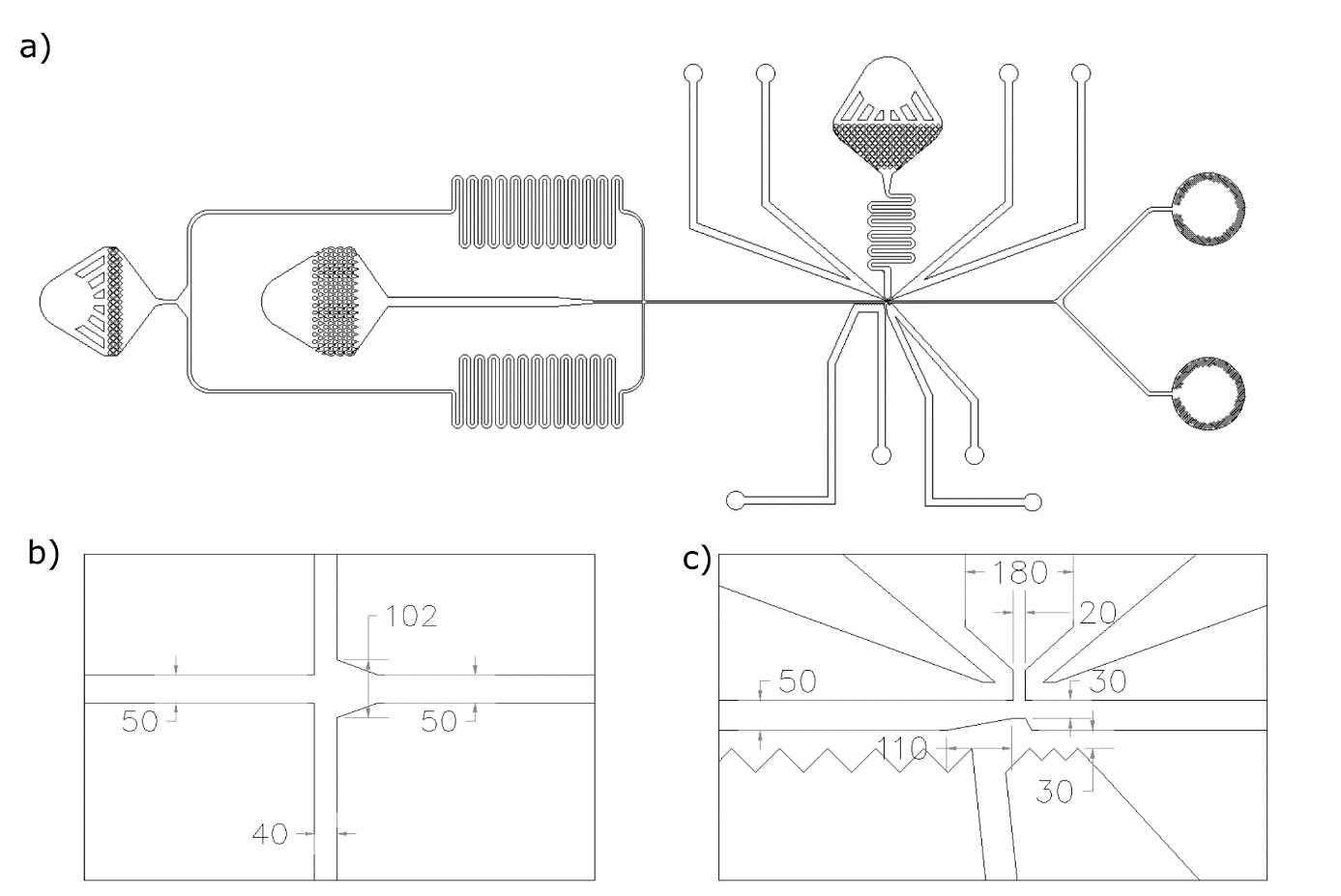


Fig. S25. Design of droplet picoinjection chip. a) Overview image showing fluidic channels and electrode structures. b) Zoom-in image showing channel dimensions at droplet reinjection nozzle. c) Zoom-in image showing channel dimensions at droplet picoinjection nozzle. All annotations are in micrometer unit.


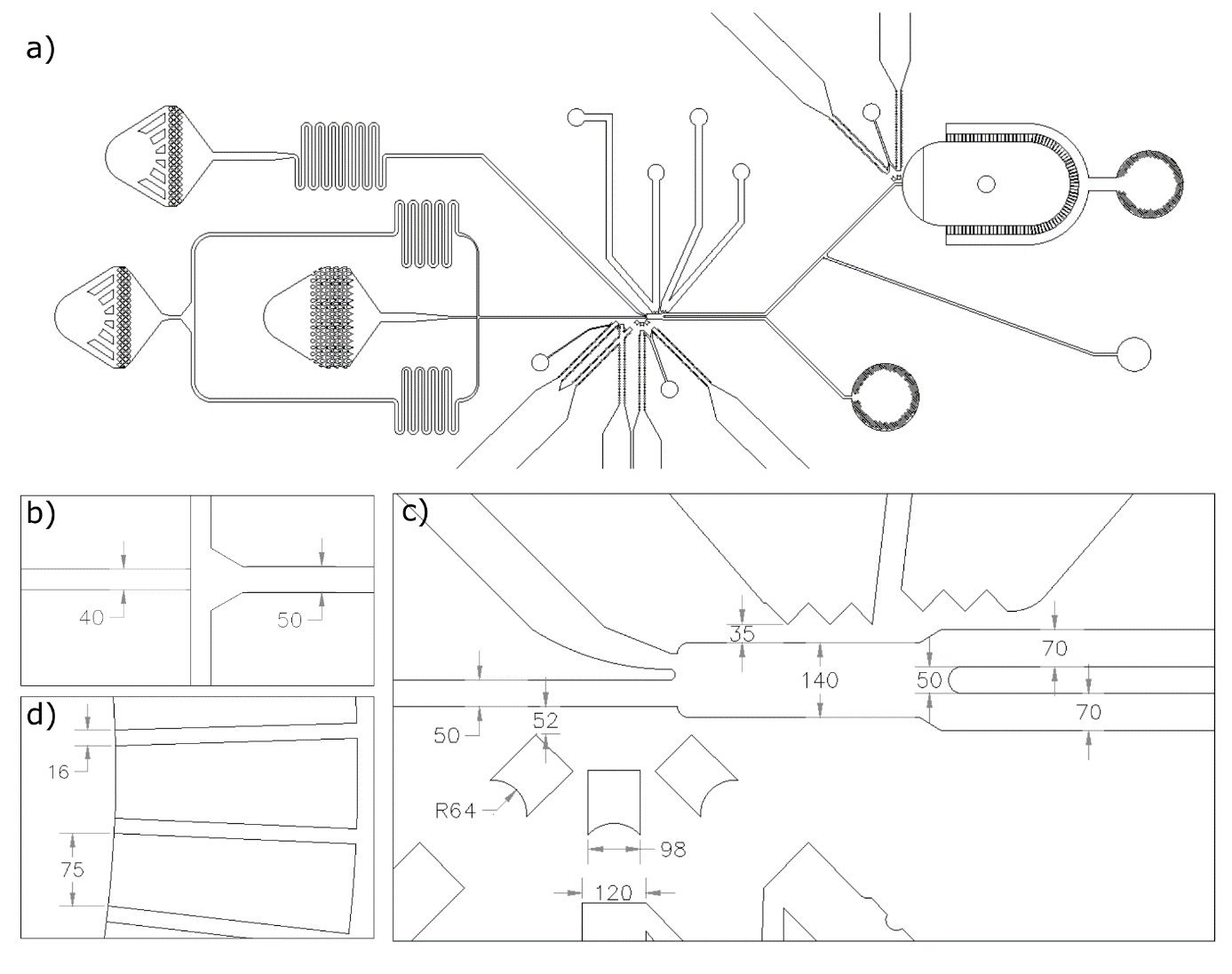


Fig. S26. Design of droplet sorting chip. a) Overview image showing fluidic channels, electrode structures and optical fiber guide structures. b) Zoom-in image showing channel dimensions at droplet reinjection nozzle. c) Zoom-in image showing channel dimensions at droplet sorting region, along with fiber guide structures and electrodes. d) Zoom-in image from the outer border of collection chamber showing thin channels through which oil phase passes while droplets are trapped. All annotations are in micrometer unit.

# References

1. Gräfe, U., Bocker, H. & Thrum, H. Regulative influence of o-aminobenzoic acid on the biosynthesis of nourseothricin in cultures of *Streptomyces noursei* JA 3890b IV. Bistability of metabolism and the mechanism of action of aminobenzoic acids. *Z. Allg. Mikrobiol.* **19**, 235–246 (2007).

2. Schroeder, I. D. R., Lam, S., Jacqueline, M., Haven, E. & Hesler, G. A. Staurosporine fermentation process. vol. 9 1–4 (1990).

3. Wolf, H., Zähner, H. & Nierhaus, K. Kirromycin, an inhibitor of the 30 S ribosomal subunits function. *FEBS Lett.* **21**, 347–350 (1972).

4. Schatz, A., Bugle, E. & Waksman, S. A. Streptomycin, a substance exhibiting antibiotic activity against Gram-positive and Gram-negative bacteria.*. *Exp. Biol. Med.* **55**, 66–69 (1944).

5. Hata, T. *et al.* Studies on the antitumor activity of an alazopeptin isolated from a new strain of *Streptomyces*. *J. Antibiot. (Tokyo).* **26**, 181–183 (1973).

6. Krügel, H., Fiedler, G., Smith, C. & Baumberg, S. Sequence and transcriptional analysis of the nourseothricin acetyltransferase-encoding gene nat1 from *Streptomyces noursei*. *Gene* **127**, 127–131 (1993).

7. Weber, T. *et al.* Recovery and isolation of individual microfluidic picoliter droplets by triggered deposition. *Sensors Actuators B Chem.* **369**, 132289 (2022).

8. Mahler, L. *et al.* Enhanced and homogeneous oxygen availability during incubation of microfluidic droplets. *RSC Adv.* **5**, 101871–101878 (2015).
